# Relativistic Quantum Metrology Beyond the X-State Ansatz: Single- and Two-Qubit Estimation of Phase and Charging Time

**DOI:** 10.3390/e28101144

**Published:** 2026-10-17

**Authors:** Samira Ebrahimi AslMamaghani, Hossein Rangani Jahromi, Rosario Lo Franco

**Affiliations:** 1 Dipartimento di Ingegneria, Università di Palermo, Viale delle Scienze, 90128 Palermo, Italy; samira.ebrahimiaslmamaghani@unipa.it; 2 Physics Department, Faculty of Sciences, Jahrom University, Jahrom P.O. Box 74135111, Iran

**Keywords:** relativistic quantum metrology, quantum battery, quantum Fisher information, Unruh–DeWitt detector, non-X quantum states, quantum parameter estimation, entanglement, concurrence, Cramér–Rao bound

## Abstract

Relativistic motion introduces decoherence that limits quantum parameter estimation. Earlier treatments of bipartite Unruh–DeWitt detectors have often restricted the reduced state to the X-state form. We consider single-qubit and isolated-ancilla two-qubit probes whose reduced state is not restricted to this form, moving along a trajectory that combines uniform acceleration with a transverse velocity. The quantum Fisher information for an initially encoded phase and for a charging time across the three velocity regimes is investigated. The two parameters respond to acceleration in distinct ways, and the isolated ancilla improves both estimations over different portions of the parameter space. Phase estimation remains nearly unaffected by the transverse velocity of the probe, whereas at short drive durations, the charging-time QFI reverses with acceleration. The separable regime that follows entanglement loss is not devoid of metrological value: both parameters can still be estimated, and the discord of the reduced state remains nonzero. These results point to quantum correlations beyond entanglement as a resource for parameter estimation in accelerated probes.

## 1. Introduction

Quantum metrology is dedicated to achieving the ultimate precision in estimating physical parameters by using quantum mechanical resources [1,2,3,4,5,6,7,8,9]. The theoretical foundation of this field is the quantum Cramér–Rao bound, which provides a fundamental lower limit on the variance of any unbiased estimator [10,11]. This variance is inversely bounded by the quantum Fisher information, which is a rigorous measure of the distinguishability of a quantum state under infinitesimal parameter variations [12]. In realistic implementations, sensors are inevitably coupled to their environments, leading to non-unitary open system dynamics. Such interactions induce decoherence and dissipation, which generally degrade metrological precision [13]. Consequently, analyzing the behavior of the quantum Fisher information in noisy environments is important for the development of robust quantum technologies.

As quantum technologies advance, understanding parameter estimation in regimes where relativistic effects cannot be ignored has become increasingly necessary, giving rise to the field of relativistic quantum information [14]. Relativistic quantum metrology [15,16,17] investigates how spacetime geometry, observer motion, and acceleration affect the fundamental limits of precision [18]. The standard framework for these theoretical explorations is the Unruh–DeWitt detector model [19], which describes a localized quantum system—often modeled as a two-level atom or qubit—interacting with a relativistic quantum field [20,21,22]. When such a detector undergoes acceleration, it perceives the Minkowski vacuum as a thermal bath—a phenomenon known as the Unruh effect [23,24]. This interaction causes the detector to thermalize and lose its initial quantum coherence without requiring a traditional material environment [25]. Recent studies have extensively employed accelerating Unruh–DeWitt detectors as moving quantum sensors to estimate Unruh temperatures and field couplings, revealing complex dependencies between relativistic trajectories and metrological sensitivity [26,27,28,29,30,31,32,33].

A crucial distinction in parameter estimation theory lies between static preparation parameters and dynamical process parameters. Static parameters, such as the initial phase of a prepared quantum state, are encoded before any temporal evolution occurs. As the system interacts with the environment, the precision with which this phase can be estimated typically undergoes monotonic degradation because the distinguishing coherences are irreversibly lost to decoherence [34,35,36]. Conversely, dynamical parameters, such as the total charging time or interaction duration, are continuously imprinted on the system during its evolution [37,38]. Estimating dynamical parameters introduces a fundamental competition: extending the interaction time allows the probe to gather more information about the process but simultaneously subjects it to prolonged environmental noise. Clarifying how relativistic effects uniquely alter the quantum Fisher information for preparation versus dynamical parameters remains an important objective for optimizing moving quantum sensors.

To mitigate noise and enhance sensitivity, contemporary metrological schemes frequently employ bipartite or ancilla-assisted probe architectures that rely on quantum entanglement [39,40,41,42,43,44,45]. A typical configuration involves a bipartite probe where an ancillary qubit, completely shielded from the environment, is entangled with the primary interacting qubit [46]. The degree of entanglement in such two-qubit systems is typically quantified by the concurrence [47]. In entanglement-assisted metrology, the relation between entanglement and metrological sensitivity depends on the probe state, the parameter encoding, and the estimation protocol [48]. Quantum correlations beyond entanglement can also contribute to metrological performance [49,50]. However, quantum correlations that do not require entanglement, commonly quantified by quantum discord [51,52], can also serve as a metrological resource [49,50], and the exact relationship between entanglement, the residual correlations of separable states, and the local thermalization induced by Unruh radiation remains highly non-trivial [53,54,55]. Determining whether entanglement universally guarantees a quantum advantage under extreme relativistic conditions is a major focus of current theoretical research.

Several analytical studies of bipartite relativistic quantum systems employ the X-state form [56,57], for which the density matrix contains nonzero elements only on the main diagonal and anti-diagonal. Related X-state reductions arise naturally in studies of accelerating Unruh–DeWitt detectors, particularly for their equilibrium or stationary reduced states [58,59]. Within these settings, accelerated X-state systems have displayed parameter-dependent Fisher-information behavior, including intervals over which the Fisher information remains constant as the Unruh acceleration varies [57]. Relativistic phase estimation has been related to maximal steered coherence, and a compact expression of that measure has been obtained for general X states [17]. In accelerating Unruh–DeWitt detectors, coherence dynamics have also been connected to quantum Fisher information for Unruh-temperature estimation [58]. More recently, phase-encoded X states have been reported to retain finite phase quantum Fisher information after entanglement sudden death [60]. The phenomenon of entanglement sudden death in *X* states and its dependence on environmental parameters have been studied extensively for two-qubit systems [61,62]. For a driven two-qubit probe coupled to a relativistic field, the reduced state can instead take a general non-X form, which allows the metrological response of the full reduced state to be examined without imposing the X-state restriction.

In this paper, we investigate the quantum Fisher information of a relativistically evolving Unruh–DeWitt detector for a two-qubit probe whose reduced dynamics is not restricted to the X-state form. The detector travels along a trajectory featuring both uniform four-acceleration and a constant transverse four-velocity component, allowing us to explore the non-relativistic, relativistic, and ultra-relativistic regimes. Within this weak-coupling Markovian framework, we obtain an analytic solution for the reduced detector dynamics and evaluate the metrological precision for two distinct parameters: the initial phase (a static preparation parameter) and the charging time (a dynamical process parameter).

We examine both a single-qubit probe and a composite two-qubit probe in which one qubit interacts with the field and the other remains isolated, and we evaluate the quantum Fisher information for the two parameters across the three velocity regimes. We also investigate the role of quantum correlations in parameter estimation by comparing the QFI with the concurrence and with quantum discord. Our analysis shows that the behavior of the two parameters is not uniform. The phase QFI decreases with acceleration in a manner similar to the concurrence, while the charging-time QFI follows a different behavior at short drive durations. At intermediate accelerations, the two-qubit probe provides a higher phase QFI than the single-qubit probe, the advantage shifting toward smaller accelerations at longer drive durations. For charging-time estimation, the ancilla improves the precision with the gain concentrated at low accelerations for long drive durations and extending over a wider interval for short ones. For short charging times, the dependence of the charging-time QFI on the transverse velocity reverses with acceleration: it decreases with transverse velocity at small accelerations and increases at larger ones. In all cases, the QFI saturates at large transverse velocities. Moreover, we found that the discord of the reduced state remains nonzero in the separable regime where the QFIs are still finite. These results show the different roles played by entanglement and discord in relativistic parameter estimation and identify a regime in which correlations other than entanglement accompany the residual metrological sensitivity of the probe.

The paper is organized as follows. In Section 2, we outline the foundational tools of our analysis, reviewing the quantum Fisher information, the quantum Fisher information matrix, and the quantum correlation measures used to characterize the two-qubit probe: concurrence, entropic discord, and geometric discord. In Section 3, we define the Unruh–DeWitt detector model, specify the relativistic trajectory, and present the master equations governing both the single-qubit and isolated-ancilla two-qubit dynamics. Section 4 presents our main results, detailing the behavior of the quantum Fisher information for both parameters across the three velocity regimes. Section 5 examines the roles of entanglement and quantum discord in parameter estimation, and it identifies the regime in which the QFIs remain finite after entanglement has vanished. Finally, we provide a summary of our findings and conclude in Section 6.

## 2. Preliminaries

### 2.1. Quantum Fisher Information

In quantum parameter estimation, the ultimate precision achievable for an unknown parameter λ encoded in a quantum state ρ(λ) is bounded by the quantum Cramér–Rao inequality

$$\mathrm{Var}\left(\hat{\lambda }\right)\geq \frac{1}{M\;f_{\lambda }},$$

where *M* is the number of independent measurements and $f_{\lambda }$ is the quantum Fisher information (QFI). The QFI is defined via the symmetric logarithmic derivative (SLD) $L_{\lambda }$, which satisfies

(1) $$\frac{\partial \rho (\lambda )}{\partial \lambda }=\frac{1}{2}\left(\rho \left(\lambda \right)L_{\lambda }+L_{\lambda }\;\rho \left(\lambda \right)\right),$$

and is given by

(2) $$f_{\lambda }=\mathrm{Tr}\;\left[\rho \left(\lambda \right)\;L_{\lambda }^{2}\right].$$

For a single qubit, the density matrix can be written in the Bloch representation as

$$\rho =\frac{1}{2}\left(I+\vec{r}\cdot \vec{\sigma }\right),\;\vec{r}=\left(r_{x},r_{y},r_{z}\right),\;\left|\vec{r}\right|\leq 1,$$

where $\vec{\sigma }$ denotes the vector of Pauli matrices. The QFI then takes the compact form [13]

(3) $$f_{\lambda }=\left\{\begin{array}{cc} {|\partial_{\lambda }\vec{r}|}^{2}+\frac{{\left(\vec{r}\cdot \partial_{\lambda }\vec{r}\right)}^{2}}{1-{|\vec{r}|}^{2}}, & \left|\vec{r}\right|<1,\\ |\partial_{\lambda }\vec{r}{|}^{2}, & \left|\vec{r}\right|=1. \end{array}\right.$$

A general density matrix can be spectrally decomposed as $\rho \left(\lambda \right)=\sum_{k}\theta_{k}|k\rangle \langle k|$, where $\theta_{k}$ are the eigenvalues and |k〉 the corresponding eigenvectors. On this basis, the QFI reads [2,10]

(4) $$f_{\lambda }=2\sum_{k,l}\frac{|\langle k|\;\partial_{\lambda }{\rho \left(\lambda \right)\;|l\rangle |}^{2}}{\theta_{k}+\theta_{l}},\;\theta_{k}+\theta_{l}\neq 0.$$

The off-diagonal (k≠l) terms highlight the role of quantum coherence and correlations in enhancing parameter sensitivity. If ρ(λ) is a pure state, ρ=|ψ〉〈ψ|, Equation (4) reduces to

(5) $$f_{\lambda }=4\left(\langle \partial_{\lambda }\psi |\partial_{\lambda }\psi \rangle -{\left|\langle \psi |\partial_{\lambda }\psi \rangle \right|}^{2}\right).$$

When several parameters $\lambda =(\lambda_{1},\lambda_{2},\ldots )$ are to be estimated simultaneously, the precision bounds are contained in the quantum Fisher information matrix (QFIM) with elements [63]

(6) $$H_{ij}=2\;\mathrm{Vec}{\left[\partial_{i}\hat{\rho }\right]}^{†}\;{\left(\bar{\hat{\rho }}⊗\hat{I}+\hat{I}⊗\hat{\rho }\right)}^{-1}\;\mathrm{Vec}\left[\partial_{j}\hat{\rho }\right],$$

where $\bar{\hat{\rho }}$ is the complex conjugate of $\hat{\rho }$, $\hat{I}$ is the identity operator, ⊗ denotes the Kronecker product, and Vec[·] stacks the columns of a matrix into a column vector. The diagonal element $H_{ii}$ gives the QFI for the single parameter $\lambda_{i}$. This vectorized form, which avoids an explicit diagonalization of the density matrix, is employed for the two-qubit calculations in this work, while the single-qubit QFI is computed directly from Equation (3).

### 2.2. Quantum Correlation Measures

We characterize the quantum correlations of the two-qubit probe using three distinct measures: the concurrence for entanglement and two discords for nonclassical correlations beyond entanglement. The three are formulated below in a general setting; they will be applied to the specific state $\rho^{\left(2q\right)}\left(\tau \right)$ of Equation (37) in Section 4.

#### 2.2.1. Concurrence

Entanglement in a two-qubit system is quantified by the concurrence [39,40]. For a density matrix ρ, one first constructs the spin-flipped state

(7) $$\tilde{\rho }=\left(\sigma_{y}⊗\sigma_{y}\right)\;\rho^{*}\;\left(\sigma_{y}⊗\sigma_{y}\right),$$

where $\rho^{*}$ is the complex conjugate of ρ in the computational basis and $\sigma_{y}$ is the Pauli-*Y* matrix. The non-Hermitian matrix $R=\rho \;\tilde{\rho }$ is then formed. Let $\lambda_{1}\geq \lambda_{2}\geq \lambda_{3}\geq \lambda_{4}$ be the square roots of the eigenvalues of *R*. The concurrence is

(8) $$C\left(\rho \right)=\max\left(0,\;\lambda_{1}-\lambda_{2}-\lambda_{3}-\lambda_{4}\right).$$

A vanishing concurrence C=0 signals a separable state, while C=1 corresponds to a maximally entangled state. Intermediate values describe partially entangled states. Because concurrence is monotonic under local operations and classical communication, it serves as a faithful measure for tracking entanglement dynamics in the presence of noise and relativistic motion.

#### 2.2.2. Entropic Quantum Discord

The entropic discord was introduced by Ollivier and Zurek [51], while Henderson and Vedral independently introduced the closely related measurement-based measure of classical correlations [64]. For a bipartite state $\rho^{AB}$, the discord with a measurement on *B* is defined as

(9) $$D_{B}\left(\rho^{AB}\right)=I\left(\rho^{AB}\right)-J_{B}\left(\rho^{AB}\right),$$

where the quantum mutual information is

(10) $$I\left(\rho^{AB}\right)=S\left(\rho^{A}\right)+S\left(\rho^{B}\right)-S\left(\rho^{AB}\right),$$

with $S\left(\rho \right)=-\mathrm{Tr}\left[\rho \log_{2}\rho \right]$ the von Neumann entropy in bits, and the classical correlation is

(11) $$J_{B}\left(\rho^{AB}\right)=S\left(\rho^{A}\right)-\min_{\left\{\Pi_{k}^{B}\right\}}\sum_{k}p_{k}\;S\left(\rho^{A|k}\right).$$

Here, $\left\{\Pi_{k}^{B}\right\}$ is a measurement on *B* with outcome probabilities $p_{k}=\mathrm{Tr}\left[\left(I⊗\Pi_{k}^{B}\right)\rho^{AB}\right]$ and conditional states $\rho^{A|k}={\mathrm{Tr}}_{B}\left[\left(I⊗\Pi_{k}^{B}\right)\rho^{AB}\right]/p_{k}$. The discord with measurement on *A*, denoted $D_{A}$, is obtained by exchanging the roles of *A* and *B*. For general states, $D_{A}$ and $D_{B}$ need not coincide; exchange symmetry of the state guarantees $D_{A}=D_{B}$, but it is not a necessary condition for equality.

The vanishing of the one-sided discords characterizes the states that are classical on one subsystem,

(12) $$D_{B}=0\;⟺\;\rho^{AB}\in {\mathrm{QC}}_{B},\;D_{A}=0\;⟺\;\rho^{AB}\in {\mathrm{CQ}}_{A},$$

where

(13) $${\mathrm{QC}}_{B}=\left\{\sum_{k}p_{k}\;\rho_{k}^{A}⊗|k\rangle {\langle k|}_{B}\right\},\;{\mathrm{CQ}}_{A}=\left\{\sum_{k}p_{k}\;|k\rangle {\langle k|}_{A}⊗\rho_{k}^{B}\right\},$$

with $p_{k}\geq 0$, $\sum_{k}p_{k}=1$, each $\rho_{k}$ a density operator, and the basis {|k〉} ranging over all orthonormal bases of the corresponding subsystem. The states that are classical in both directions form the intersection $\mathrm{CC}={\mathrm{CQ}}_{A}\cap {\mathrm{QC}}_{B}$.

For a two-qubit state, the optimization in Equation (11) can be parametrized in terms of the Bloch representation. Writing

(14) $$\rho^{AB}=\frac{1}{4}\left(I⊗I+\vec{x}\cdot \vec{\sigma }⊗I+I⊗\vec{y}\cdot \vec{\sigma }+\sum_{i,j=1}^{3}T_{ij}\;\sigma_{i}⊗\sigma_{j}\right),$$

with Bloch vectors $\vec{x},\vec{y}$ and correlation matrix *T*, a rank-one projective measurement on *B* along the unit vector $\hat{n}=\left(\sin\xi \cos\zeta ,\;\sin\xi \sin\zeta ,\;\cos\xi \right)$ is described by the projectors

(15) $$\Pi_{\pm }^{B}=\frac{1}{2}\left(I\pm \hat{n}\cdot \vec{\sigma }\right).$$

These produce outcomes with probabilities

(16) $$p_{\pm }=\frac{1\pm \vec{y}\cdot \hat{n}}{2}$$

and conditional Bloch vectors

(17) $${\vec{x}}_{\pm }=\frac{\vec{x}\pm T\hat{n}}{1\pm \vec{y}\cdot \hat{n}},$$

defined for $p_{\pm }>0$; when $p_{\pm }=0$, the corresponding outcome is omitted from the conditional-entropy sum. The conditional entropy entering Equation (11) is therefore $S\left(\rho^{A|\pm }\right)=h_{2}\left[(1+\right|{\vec{x}}_{\pm }\left|)/2\right]$, where $h_{2}\left(u\right)=-u\log_{2}u-\left(1-u\right)\log_{2}\left(1-u\right)$ is the binary entropy. The minimization reduces to a two-dimensional search over (ξ,ζ) on the Bloch sphere.

##### Optimization

In the present work, the minimization in Equation (11) is performed over projective (von Neumann) measurements, following the original definition of Ollivier and Zurek [51]. The resulting quantities $D_{A}$ and $D_{B}$ are therefore projective-measurement discords—not full positive-operator-valued-measure (POVM) discords. For two-qubit states of rank two, von Neumann measurements are optimal [65], so the distinction is immaterial in that case. For some two-qubit states of rank larger than two, three-element POVMs can yield a smaller conditional entropy than any von Neumann measurement [65]. The state $\rho^{\left(2q\right)}\left(\tau \right)$ of Equation (37) is generally not restricted to rank two, so we report $D_{A}$ and $D_{B}$ as projective-measurement discords throughout. The numerical optimization uses a grid search over the Bloch-sphere angles (ξ,ζ) followed by a local refinement, including the poles ξ=0 and ξ=π to avoid missing global minima that lie on the measurement axis.

#### 2.2.3. Geometric Quantum Discord

The geometric discord of Dakić, Vedral, and Brukner [52] is defined as the squared Hilbert–Schmidt distance from $\rho^{AB}$ to the closest classical–quantum state,

(18) $$D_{G}^{A}\left(\rho^{AB}\right)=\min_{\chi \in {\mathrm{CQ}}_{A}}{∥\rho^{AB}-\chi ∥}_{2}^{2},$$

where ${\mathrm{CQ}}_{A}$ is the set defined in Equation (13), and ${∥M∥}_{2}=\sqrt{\mathrm{Tr}[M^{†}M]}$ denotes the Hilbert–Schmidt (Frobenius) norm of a matrix *M*, with ${∥\vec{v}∥}^{2}=\sum_{i}v_{i}^{2}$ the squared Euclidean norm of a vector $\vec{v}$. For two-qubit states, the minimization admits the closed-form solution [52]

(19) $$D_{G}^{A}\left(\rho^{AB}\right)=\frac{1}{4}\left({∥\vec{x}∥}^{2}+{∥T∥}_{2}^{2}-\lambda_{\max}\;\left(\vec{x}\;{\vec{x}}^{T}+T\;T^{T}\right)\right),$$

where $\lambda_{\max}$ is the largest eigenvalue of the 3×3 matrix $\vec{x}{\vec{x}}^{T}+TT^{T}$, and $\vec{x}$ and *T* are the Bloch vector and correlation matrix appearing in Equation (14). Equation (19) evaluates the *A*-side geometric discord, that is, the classical subsystem is *A*; the *B*-side counterpart follows from the replacements $\vec{x}\to \vec{y}$ and $T\to T^{T}$. The factor 1/4 is the unrescaled convention of Ref. [52]. With this convention, a Bell state gives $D_{G}^{A}=1/2$, whereas the entropic discords evaluated in bits give $D_{A}=D_{B}=1$. The three quantities are distinct functionals of the same state and should not be identified numerically. The geometric discord requires no numerical optimization over measurements: Equation (19) is evaluated directly from the Bloch decomposition of $\rho^{\left(2q\right)}\left(\tau \right)$.

##### Relation Among the Three Measures

The three quantities $D_{A}$, $D_{B}$, and $D_{G}^{A}$ are distinct functionals of the same state. Under the convention in which $D_{A}$ and $D_{B}$ denote the entropic discords with projective measurements on *A* and *B*, respectively, their vanishing conditions are those of Equation (12), and because $D_{G}^{A}$ is the squared distance to ${\mathrm{CQ}}_{A}$, it vanishes precisely on the same set as $D_{A}$. The simultaneous vanishing of all three quantities therefore occurs for classical–classical states,

(20) $$D_{A}=D_{B}=D_{G}^{A}=0\;⟺\;\rho^{AB}\in \mathrm{CC}.$$

We do not use any general inequality among these correlation measures in the present analysis.

## 3. Model

We consider a two-level quantum system—a quantum battery—moving along a spatial trajectory in flat (3 + 1)-dimensional Minkowski spacetime, which is confined to a two-dimensional plane [24]. The battery is modeled as an Unruh–DeWitt detector: a localized qubit that couples to a massless scalar field. Although we use the terms “battery” and “charging time,” the present investigation focuses on quantum metrology rather than thermodynamics. The “charging time” denotes the duration of the charging protocol, that is, the interval during which the external drive is active, and it acts as the parameter to be estimated.

Units and Conventions

We use natural units with c=ℏ=1. Time and length then share the same dimension, *T*, and the quantities appearing in the manuscript have the following dimensions:

$$\left[s\right]=\left[\tau \right]=T,\;\left[x^{\mu }\right]=T,$$

$$\left[a\right]=\left[\alpha \right]=\left[\Omega \right]=\left[A\right]=\left[B\right]=\left[C\right]=T^{-1},$$

$$\left[w\right]=\left[\lambda \right]=\left[\theta \right]=\left[\phi \right]=\left[f_{\phi }\right]=\left[R\right]=1,\;\left[f_{\tau }\right]=T^{-2}.$$

We use *s* for the proper time along the trajectory and τ for the total charging time. The Wightman function is written as $G^{+}\left(\Delta s\right)$ with $\Delta s=s-s^{\prime }$. In the iϵ prescription of Equation (25), ϵ is a formal infinitesimal that is taken to zero and does not represent a physical parameter of the model.

The numerical values in the figures are expressed in these natural units. An absolute SI interpretation would require a physical frequency or energy scale, which the present study does not assign.

Detector Trajectory

The battery follows a non-inertial worldline $x\left(s\right)=(t\left(s\right),\vec{x}\left(s\right))$ with uniform proper acceleration along the *x*-direction and a constant four-velocity component in the *y*-direction. The trajectory is parameterized by the proper time *s*:

(21) $$x\left(s\right)=\left(\frac{a}{\alpha^{2}}\sinh\left(\alpha s\right),\;\frac{a}{\alpha^{2}}\cosh\left(\alpha s\right),\;ws,\;0\right),$$

where a>0 is the invariant magnitude of the four-acceleration, w=dy/ds is the *y*-component of the four-velocity, and $\alpha =a/\sqrt{1+w^{2}}$ is a positive constant. The four-velocity $u^{\mu }=dx^{\mu }/ds$ satisfies $u^{\mu }u_{\mu }=-1$ (natural units c=ℏ=1), and the four-acceleration $a^{\mu }=d^{2}x^{\mu }/ds^{2}$ has the invariant magnitude $a=\sqrt{a^{\mu }a_{\mu }}$. This motion combines hyperbolic acceleration along the *x*-axis with a constant four-velocity component along the *y*-axis; there is no acceleration in the *y* or *z* directions.

Hamiltonian of the Combined System

The battery is modeled as an Unruh–DeWitt detector that couples to a massless scalar field Φ(x). The resonant driving term is written in the rotating frame after the rotating-wave approximation for the classical drive, while the detector-field interaction is retained in the Unruh–DeWitt form. The effective Hamiltonian for the reduced dynamics is

(22) $$H=H_{d}+H_{f}+H_{\mathrm{int}},$$

where the following apply:

- $H_{d}=\mu \left(s\right)\;\frac{\Omega }{2}\left(\sigma^{+}+\sigma^{-}\right)$ represents the driving (charging) Hamiltonian, describing the interaction of the two-level battery with an external classical field. The switching function μ(s) equals 1 for 0≤s≤τ and 0 otherwise. The charging time τ is the duration of the drive. The external field is resonant with the detector transition, so its carrier frequency matches the detector’s natural transition frequency $\omega_{0}$, where $H_{0}=\omega_{0}\sigma^{+}\sigma^{-}$ is the free detector Hamiltonian. In the effective rotating-frame description, Ω denotes the coherent charging amplitude (effective Rabi frequency). We set $\Omega =\omega_{0}$ as a simplifying parameter choice in the present model. The operators $\sigma^{+}$ and $\sigma^{-}$ are the raising and lowering operators:
  $$\sigma^{+}=|e\rangle \langle g|,\;\sigma^{-}=|g\rangle \langle e|.$$
  They satisfy
  $$\sigma_{x}=\sigma^{+}+\sigma^{-},\;\sigma_{y}=i\left(\sigma^{+}-\sigma^{-}\right),\;\sigma_{z}=\left|g\rangle \langle g\right|-|e\rangle \langle e|,$$
  and
  $$\left[\sigma^{+},\sigma^{-}\right]=-\sigma_{z}.$$
- $H_{f}$ is the free Hamiltonian of the scalar field in Minkowski spacetime.
- $H_{\mathrm{int}}=\lambda \left(\sigma^{+}+\sigma^{-}\right)\;\Phi \left(x\left(s\right)\right)$ is the interaction between the detector and the field with coupling constant λ.

Master Equation and Decay Parameters

Under the weak-coupling assumption $\lambda^{2}\ll 1$, the reduced dynamics of the detector is governed by a Kossakowski–Lindblad master equation:

(23) $$\frac{\partial }{\partial \tau }\;\rho \left(\tau \right)=-i\left[H_{\mathrm{eff}}^{\left(d\right)},\rho \left(\tau \right)\right]+\frac{1}{2}\sum_{i,j=1}^{3}a_{ij}\;D_{ij}\left[\rho \left(\tau \right)\right],$$

where ρ(τ) is the reduced density matrix of the detector and τ runs from 0 to the total charging duration. The state ρ(τ) is evaluated at the end of the drive, where τ equals the charging time. The dissipator is defined as

$$D_{ij}\left(\rho \right)=2\sigma_{j}\rho \;\sigma_{i}-\sigma_{i}\sigma_{j}\rho -\rho \;\sigma_{i}\sigma_{j}.$$

The coefficients $a_{ij}$ are given by

(24) $$a_{ij}=A\delta_{ij}-iB\epsilon_{ijk}\delta_{k1}+C\delta_{i1}\delta_{j1},$$

with *A*, *B*, *C* real constants determined from the field correlation function along the detector’s trajectory. The effective detector Hamiltonian is

$$H_{\mathrm{eff}}^{\left(d\right)}=\frac{\Omega^{\prime }}{2}\left(\sigma^{+}+\sigma^{-}\right),\;\Omega^{\prime }=\Omega +\delta \Omega ,$$

where δΩ is the Lamb shift. The dissipative coefficients *A*, *B*, and *C* are of order $\lambda^{2}$, and the weak-coupling condition $\lambda^{2}\ll 1$ is satisfied in the present study. In the numerical treatment, we neglect the Lamb shift and set $\Omega^{\prime }=\Omega$. This is a simplifying approximation of the present work and is not derived from $\lambda^{2}\ll 1$ alone.

Field Correlation Function and Decay Coefficients

Define the vacuum Wightman function along the trajectory:

$$G^{+}\left(\Delta s\right)=\langle 0|\Phi \left(x\left(s\right)\right)\Phi \left(x\left(s^{\prime }\right)\right)|0\rangle ,\;\Delta s=s-s^{\prime }.$$

For the trajectory given above, this evaluates to

(25) $$G^{+}\left(\Delta s\right)=-\frac{\alpha^{4}}{16\pi^{2}a^{2}}{\left[\sinh^{2}\;\left(\frac{\alpha \Delta s}{2}-\frac{i\epsilon \alpha^{2}}{a}\right)-\frac{w^{2}\alpha^{4}}{4a^{2}}{\left(\Delta s\right)}^{2}\right]}^{-1},$$

where the iϵ prescription (with $\epsilon \to 0^{+}$) regularizes the integration. The Fourier transform and Hilbert transform of $G^{+}$ are

$$G\left(\Omega \right)=\int_{-\infty }^{\infty }d\Delta s\;e^{i\Omega \Delta s}\;G^{+}\left(\Delta s\right),\;K\left(\Omega \right)=\frac{P}{\pi i}\int_{-\infty }^{\infty }d\omega \;\frac{G(\omega )}{\omega -\Omega },$$

with P denoting the Cauchy principal value. The decay parameters are then

(26) $$\begin{array}{cc} A & =\frac{\lambda^{2}}{2}\left[G(\Omega )+G(-\Omega )\right],\\ B & =\frac{\lambda^{2}}{2}\left[G(\Omega )-G(-\Omega )\right],\;C=\lambda^{2}G\left(0\right)-A. \end{array}$$

Physical Regimes

Three regimes are distinguished by the magnitude of the dimensionless four-velocity component *w*. The transverse coordinate velocity at s=0 is

$$v_{y}\left(0\right)=\frac{w\;c}{\sqrt{1+w^{2}}},$$

which is always below *c* and approaches *c* as w→∞. We classify the regimes as follows:

- **Non-relativistic regime** (w≪1): the transverse coordinate velocity is small compared with *c*.
- **Relativistic regime** (w∼1): the transverse coordinate velocity is a finite fraction of *c*.
- **Ultra-relativistic regime** (w≫1): the transverse coordinate velocity approaches *c* from below.

These labels refer to the transverse motion controlled by *w*; the motion in the *x*-direction remains uniformly accelerated in all three regimes. In all regimes, the decay coefficients are obtained from (26) by evaluating the integrals with the appropriate form of $G^{+}\left(\Delta s\right)$.

### 3.1. Bloch Equations for the Single Qubit

Any single-qubit state can be written in the Bloch representation:

$$\rho \left(\tau \right)=\frac{1}{2}\left(I+r(\tau )\cdot \sigma \right),\;r\left(\tau \right)={\left(r_{x},r_{y},r_{z}\right)}^{T},\;\left|r\right|\leq 1,$$

where $\sigma =(\sigma_{x},\sigma_{y},\sigma_{z})$. Inserting this into the Master Equation (23) yields a system of differential equations for the Bloch vector [24]:

(27) $$\frac{dr(\tau )}{d\tau }=-2\;H\;r\left(\tau \right)+\chi ,$$

with

$$H=\left(\begin{array}{ccc} 2A & 0 & 0\\ 0 & 2A+C & \Omega /2\\ 0 & -\Omega /2 & 2A+C \end{array}\right),\;\chi =\left(\begin{array}{c} -4B\\ 0\\ 0 \end{array}\right).$$

Explicitly,

$$\begin{array}{cc} {\dot{r}}_{x}\left(\tau \right) & =-4A\;r_{x}\left(\tau \right)-4B,\\ {\dot{r}}_{y}\left(\tau \right) & =-2\left[\left(2A+C\right)r_{y}\left(\tau \right)+\frac{\Omega }{2}r_{z}\left(\tau \right)\right],\\ {\dot{r}}_{z}\left(\tau \right) & =-2\left[-\frac{\Omega }{2}r_{y}\left(\tau \right)+\left(2A+C\right)r_{z}\left(\tau \right)\right]. \end{array}$$

#### Initial State and Explicit Solution

The initial state of the single-qubit battery is chosen as

(28) $$|\psi_{0}\rangle =\sin\;\left(\frac{\theta }{2}\right)|0\rangle +e^{i\phi }\cos\;\left(\frac{\theta }{2}\right)|1\rangle ,$$

where |0〉≡|g〉 and |1〉≡|e〉. The corresponding initial Bloch components are $r_{i}\left(0\right)=\langle \psi_{0}|\sigma_{i}|\psi_{0}\rangle$, i.e.,

$$r_{x}\left(0\right)=\sin\theta \cos\phi ,\;r_{y}\left(0\right)=\sin\theta \sin\phi ,\;r_{z}\left(0\right)=-\cos\theta .$$

Solving (27) with these initial conditions gives

(29) $$\begin{array}{cc} r_{x}\left(\tau \right) & =\frac{B\left(e^{-4A\tau }-1\right)}{A}+e^{-4A\tau }\sin\theta \;\cos\phi , \end{array}$$

(30) $$\begin{array}{cc} r_{y}\left(\tau \right) & =e^{-2(2A+C)\tau }\left[\cos\theta \;\sin(\Omega \tau )+\sin\theta \;\sin\phi \;\cos(\Omega \tau )\right], \end{array}$$

(31) $$\begin{array}{cc} r_{z}\left(\tau \right) & =e^{-2(2A+C)\tau }\left[\sin\theta \;\sin\phi \;\sin(\Omega \tau )-\cos\theta \;\cos(\Omega \tau )\right]. \end{array}$$

The density matrix at time τ follows from $\rho \left(\tau \right)=\frac{1}{2}\left(I+r\left(\tau \right)\cdot \sigma \right)$:

(32) $$\rho \left(\tau \right)=\left(\begin{array}{cc} \rho_{11}\left(\tau \right) & \rho_{12}\left(\tau \right)\\ \rho_{21}\left(\tau \right) & \rho_{22}\left(\tau \right) \end{array}\right),$$

with

(33) $$\begin{array}{cc} \rho_{11}\left(\tau \right) & =\frac{1}{2}\left[1+e^{-2(2A+C)\tau }\left(\sin\theta \;\sin\phi \;\sin(\Omega \tau )-\cos\theta \;\cos(\Omega \tau )\right)\right],\\ \rho_{22}\left(\tau \right) & =\frac{1}{2}\left[1-e^{-2(2A+C)\tau }\left(\sin\theta \;\sin\phi \;\sin(\Omega \tau )-\cos\theta \;\cos(\Omega \tau )\right)\right],\\ \rho_{12}\left(\tau \right) & =\frac{1}{2}(\frac{B\left(e^{-4A\tau }-1\right)}{A}-i\;e^{-2(2A+C)\tau }\left[\cos\theta \;\sin(\Omega \tau )+\sin\theta \;\sin\phi \;\cos(\Omega \tau )\right]\\ \; & \;\;+e^{-4A\tau }\sin\theta \;\cos\phi ),\\ \rho_{21}\left(\tau \right) & =\rho_{12}^{*}\left(\tau \right). \end{array}$$

### 3.2. Two-Qubit System with an Isolated Ancilla

For the two-qubit battery, we employ the entangled initial state

(34) $$|\psi_{0}^{\left(2q\right)}\rangle =\sin\;\left(\frac{\theta }{2}\right)|00\rangle +e^{i\phi }\cos\;\left(\frac{\theta }{2}\right)|11\rangle ,$$

where only the first qubit (the battery) interacts with the scalar field, while the second (ancillary) qubit remains isolated. The two-qubit density matrix is constructed using the method of Bellomo et al. [66,67], which expresses the evolution of independent open systems from the known single-qubit dynamical map. For a single qubit *A*,

(35) $$\rho_{ii^{\prime }}^{A}\left(t\right)=\sum_{ll^{\prime }}A_{ii^{\prime }}^{ll^{\prime }}\left(t\right)\;\rho_{ll^{\prime }}^{A}\left(0\right),$$

and similarly for qubit *B*. For two independent qubits, the combined density matrix evolves as

(36) $$\rho_{ii^{\prime },jj^{\prime }}^{T}\left(t\right)=\sum_{ll^{\prime },mm^{\prime }}A_{ii^{\prime }}^{ll^{\prime }}\left(t\right)\;B_{jj^{\prime }}^{mm^{\prime }}\left(t\right)\;\rho_{ll^{\prime },mm^{\prime }}^{T}\left(0\right).$$

Applying this procedure with the single-qubit map derived from the Master Equation (23) and the corrected solution (29)–(31) yields the following two-qubit density matrix:

(37) $$\rho^{\left(2q\right)}\left(\tau \right)=\left(\begin{array}{cccc} \rho_{11} & \rho_{12} & \rho_{13} & \rho_{14}\\ \rho_{21} & \rho_{22} & \rho_{23} & \rho_{24}\\ \rho_{31} & \rho_{32} & \rho_{33} & \rho_{34}\\ \rho_{41} & \rho_{42} & \rho_{43} & \rho_{44} \end{array}\right),$$

where the nonzero elements are

(38) $$\begin{array}{cc} \rho_{11} & =\frac{1}{2}\left[1+e^{-2(C+2A)\tau }\cos\left(\Omega \tau \right)\right]\sin^{2}\left(\theta /2\right),\\ \rho_{22} & =\frac{1}{2}\left[1-e^{-2(C+2A)\tau }\cos\left(\Omega \tau \right)\right]\cos^{2}\left(\theta /2\right),\\ \rho_{33} & =\frac{1}{2}\left[1-e^{-2(C+2A)\tau }\cos\left(\Omega \tau \right)\right]\sin^{2}\left(\theta /2\right),\\ \rho_{44} & =\frac{1}{2}\left[1+e^{-2(C+2A)\tau }\cos\left(\Omega \tau \right)\right]\cos^{2}\left(\theta /2\right), \end{array}$$

(39) $$\begin{array}{cc} \rho_{13} & =\frac{e^{-2(C+2A)\tau }\sin^{2}\left(\theta /2\right)}{2A}\left[-e^{2C\tau }\left(-1+e^{4A\tau }\right)B+iA\sin\left(\Omega \tau \right)\right],\\ \rho_{24} & =\frac{e^{-2(C+2A)\tau }\cos^{2}\left(\theta /2\right)}{2A}\left[-e^{2C\tau }\left(-1+e^{4A\tau }\right)B-iA\sin\left(\Omega \tau \right)\right], \end{array}$$

(40) $$\begin{array}{cc} \rho_{12} & =\frac{i}{4}e^{-2(C+2A)\tau -i\phi }\sin\theta \;\sin\left(\Omega \tau \right),\\ \rho_{14} & =\frac{1}{4}e^{-2(C+2A)\tau -i\phi }\left(e^{2C\tau }+\cos\left(\Omega \tau \right)\right)\sin\theta ,\\ \rho_{23} & =\frac{1}{4}e^{-2(C+2A)\tau +i\phi }\left(e^{2C\tau }-\cos\left(\Omega \tau \right)\right)\sin\theta ,\\ \rho_{34} & =-\frac{i}{4}e^{-2(C+2A)\tau -i\phi }\sin\theta \;\sin\left(\Omega \tau \right), \end{array}$$

and the remaining off-diagonal entries are obtained from $\rho_{ij}=\rho_{ji}^{*}$. This density matrix is generally of non-*X* form.

## 4. Results and Metrological Analysis

### 4.1. Estimation Characteristics of Single-Qubit Systems

#### 4.1.1. Initial-Phase Estimation

The quantum Fisher information (QFI) for the phase parameter ϕ is computed using the single-qubit Bloch representation. Substituting the Bloch vector components from Equations (29)–(31) into the general expression for the QFI of a mixed qubit state [Equation (3)] gives

(41) $$\begin{array}{cc} f_{\phi } & =\frac{1}{2}\sin^{2}\theta \;e^{-8\tau (2A+C)}(e^{4\tau (2A+C)}\left(2e^{4C\tau }\sin^{2}\phi +\cos\left(2\phi \right)+1\right)\\ \; & \;+\frac{2\sin^{2}\phi {\left(B\left(e^{4A\tau }-1\right)e^{4C\tau }-A\left(e^{4C\tau }-1\right)\sin\theta \cos\phi \right)}^{2}}{A^{2}\left(1-\frac{4B^{2}e^{-4A\tau }\sinh^{2}\left(2A\tau \right)}{A^{2}}-\frac{e^{-4\tau (2A+C)}\left(e^{4C\tau }\sin\theta \cos\phi \left(A\sin\theta \cos\phi -2B\left(e^{4A\tau }-1\right)\right)+A\left(\sin^{2}\theta \;\sin^{2}\phi +\cos^{2}\theta \right)\right)}{A}\right)}). \end{array}$$

**Figure 1:**
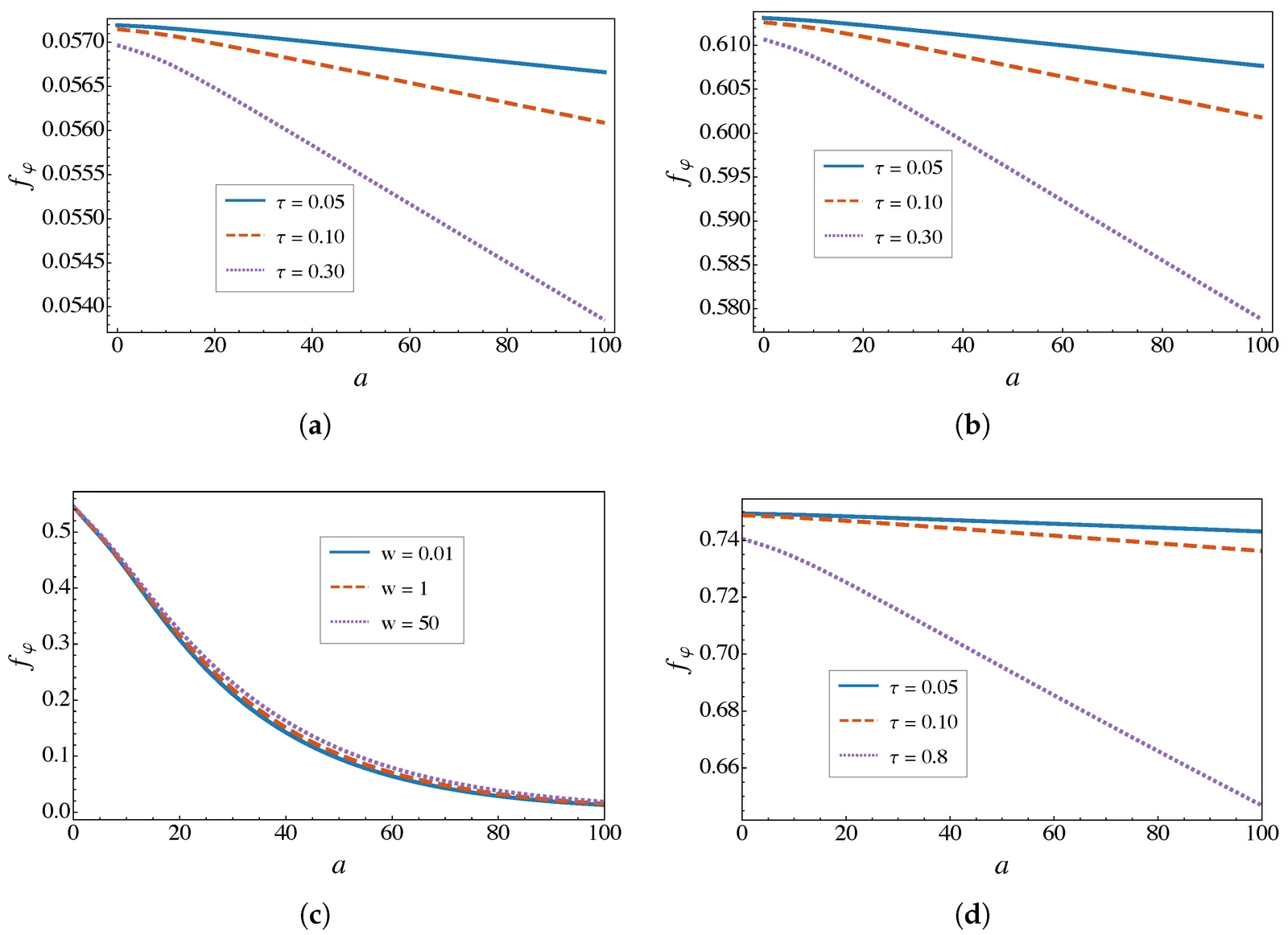
Quantum Fisher information (QFI) for phase estimation in a single-qubit quantum battery as a function of four-acceleration *a* and four-velocity component *w*. (**a**) Non-relativistic regime (w≪1), with θ=2.9, ϕ=π, and w=0.01. (**b**) Relativistic regime (w≈1), with θ=0.9, ϕ=0.7, and w=1.1. (**c**) $f_{\phi }$ versus *a* for different *w*, with $\theta_{0}=\frac{\pi }{3}$, $\phi_{0}=0.1$, λ=0.1, Ω=5, and τ=20. (**d**) Ultra-relativistic regime: $f_{\phi }$ versus *a* for different charging times τ, with $\theta_{0}=\frac{\pi }{3}$, $\phi_{0}=0.1$, λ=0.1, Ω=5, and w=100.

Figure 1a,b show the phase QFI as a function of the four acceleration of the probe for several interaction times in the non-relativistic and relativistic regimes, respectively. In both panels, $f_{\phi }$ decreases monotonically with *a*, and the decrease is larger at longer interaction times.

This dependence on *a* enters the phase QFI through the decay coefficients A(a,w,Ω) and C(a,w,Ω), obtained from the Fourier transform of the Wightman function along the trajectory. In the Bloch solution, every term that carries information about the initial phase ϕ is multiplied by either $e^{-4A\tau }$ or $e^{-2(2A+C)\tau }$ so that an increase in the field-induced decay rates reduces the phase-sensitive components of the state.

In the limit of long interaction times, the Bloch vector approaches r(τ→∞)=(−B/A,0,0), which is independent of ϕ. Consequently, $\partial_{\phi }r\to 0$ and $f_{\phi }\to 0$. The vanishing of the phase QFI in this limit is a direct consequence of the equations of motion.

The ultra-relativistic regime displays the same dependence on *a*. As shown in Figure 1c,d, the decrease in $f_{\phi }$ with *a* follows the behavior found in panels (a) and (b), and it becomes larger at longer interaction times τ. Figure 1c further shows that increasing the transverse four-velocity *w* has little effect on $f_{\phi }$, so the transverse motion of the probe does not influence the initial-phase estimation.

**Figure 2:**
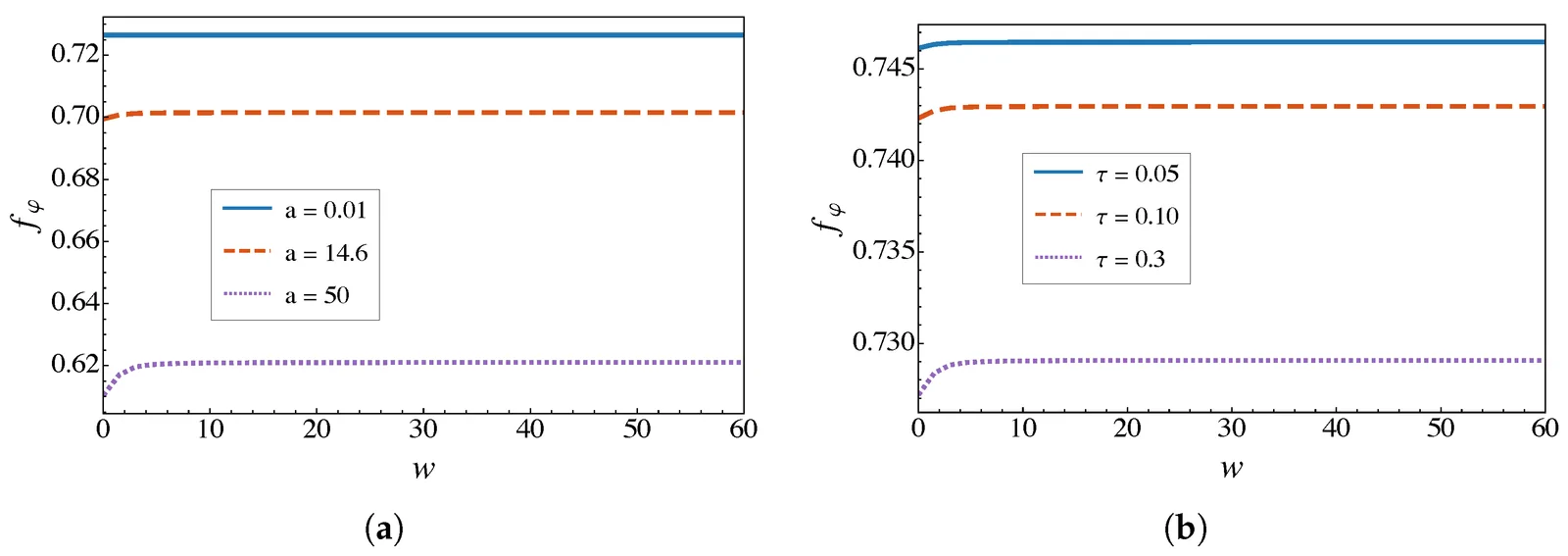
QFI for single-qubit phase estimation as a function of the four-velocity component *w*, with $\theta_{0}=\frac{\pi }{3}$, $\phi_{0}=0.1$, λ=0.1, and Ω=5. (**a**) $f_{\phi }$ versus *w* for different *a*, with τ=2. (**b**) $f_{\phi }$ versus *w* for different τ, with a=50.

Figure 2a,b verify that increasing the transverse four-velocity *w* leaves $f_{\phi }$ nearly unchanged. The QFI does increase slightly with *w*, but the enhancement is marginal and does not considerably alter the qualitative behavior. Consequently, a moving single-qubit probe approximately preserves its phase-estimation sensitivity independently of its transverse velocity, so that relativistic motion does not need to be compensated in order to maintain the precision achievable for an initially encoded phase.

#### 4.1.2. Charging Time Estimation

The QFI for the charging time τ is given by

(42) $$\begin{array}{cc} f_{\tau }= & \frac{4e^{-8\tau (2A+C)}\left[A\left(2A+C\right)X-2e^{4C\tau }Y(B\left(e^{4A\tau }-1\right)-A\sin\theta \cos\phi \right){]}^{2}}{A^{2}\left(1-\frac{4B^{2}e^{-4A\tau }\sinh^{2}\left(2A\tau \right)}{A^{2}}-\frac{e^{-4\tau (2A+C)}\left[e^{4C\tau }\sin\theta \cos\phi \left(A\sin\theta \cos\phi -2B(e^{4A\tau }-1)\right)+AX\right]}{A}\right)}\\ \; & +16e^{-8A\tau }{\left(A\sin\theta \cos\phi +B\right)}^{2}\\ \; & +e^{-4\tau (2A+C)}\left(4{\left(2A+C\right)}^{2}+\Omega^{2}\right)X. \end{array}$$

where

$$\begin{array}{c} X=\sin^{2}\theta \sin^{2}\phi +\cos^{2}\theta ,\\ Y=A\sin\theta \cos\phi +B. \end{array}$$

**Figure 3:**
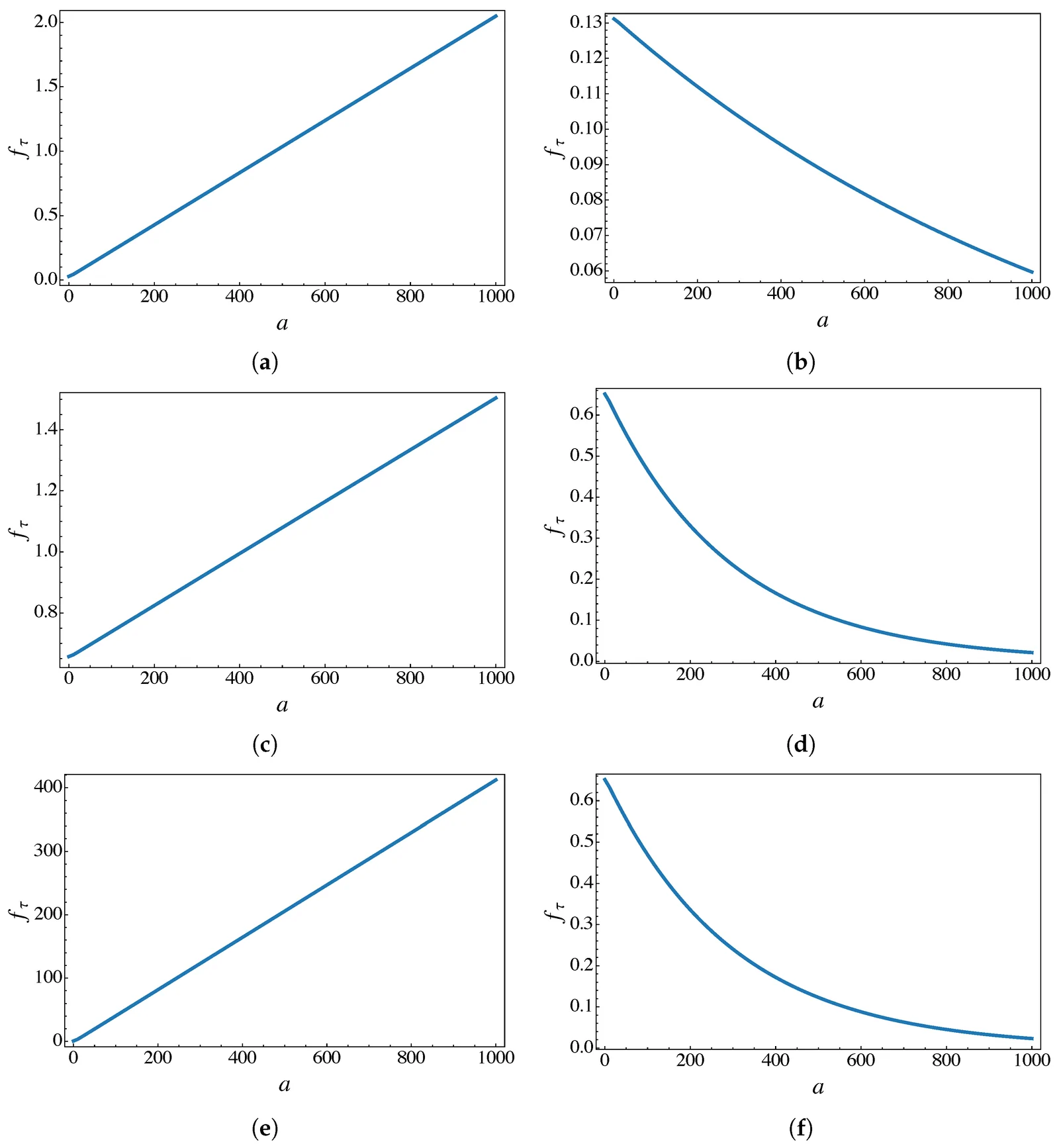
$f_{\tau }$ for a single-qubit probe as a function of four-acceleration *a* for different charging times τ and velocity regimes. Fixed parameters across regimes are ϕ=π. (**a**,**b**) Non-relativistic regime (w=0.01, Ω=1, λ=0.02) for (**a**) small τ=0.01, θ=1.4, and (**b**) large τ=10, θ=1.2. (**c**,**d**) Relativistic regime (w=1.5, Ω=1.5, θ=1, λ=0.03) for (**c**) small τ=0.05 and (**d**) large τ=20. (**e**,**f**) Ultra-relativistic regime (w=100, Ω=1.5, θ=1, λ=0.03) for (**e**) small τ=0.0001 and (**f**) large τ=20.3.

All numerical results in this subsection are obtained at ϕ=π. Figure 3 displays $f_{\tau }$ as a function of *a* in the non-relativistic, relativistic, and ultra-relativistic regimes. At short charging times, $f_{\tau }$ increases with *a* in all three regimes. As τ grows, this increase weakens and is eventually replaced by a decrease.

Acceleration affects the charging-time estimation through the trajectory-dependent rates A(a,w,Ω) and C(a,w,Ω). In contrast to phase estimation, the quantity that enters $f_{\tau }$ is the derivative $\partial_{\tau }r$, since τ determines how far the driven open-system dynamics has evolved before the state is measured. At short charging times, the dynamics is still transient, so the final state retains a finite dependence on the duration of the drive and $f_{\tau }$ changes with *a*. As τ increases, the transient contributions are suppressed. From the Bloch solution, the state approaches the stationary value $r_{\mathrm{ss}}=\left(-B/A,0,0\right)$ and $\partial_{\tau }r\to 0$ in the long-time limit. The charging-time QFI therefore vanishes. In Figure 3, the competition between the acceleration dependence of the decay rates and the finite-time evolution produces the crossover from an increase of $f_{\tau }$ with *a* at short τ to a decrease at longer τ.

**Figure 4:**
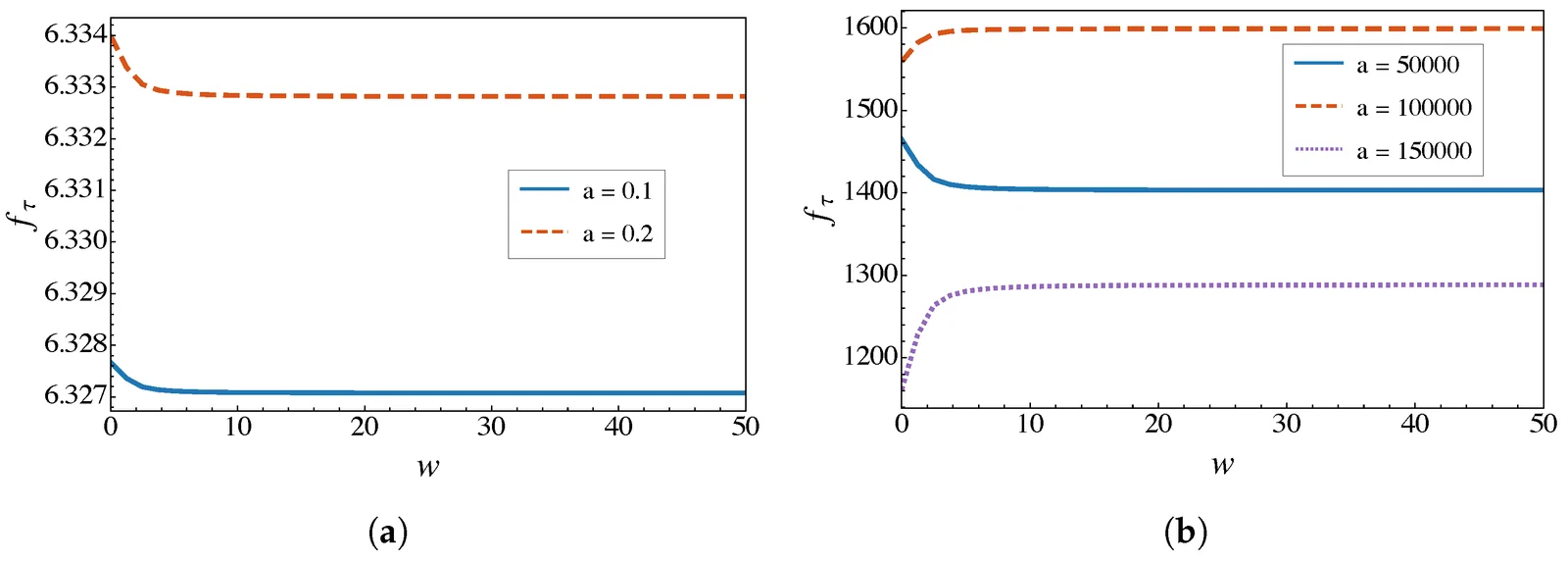

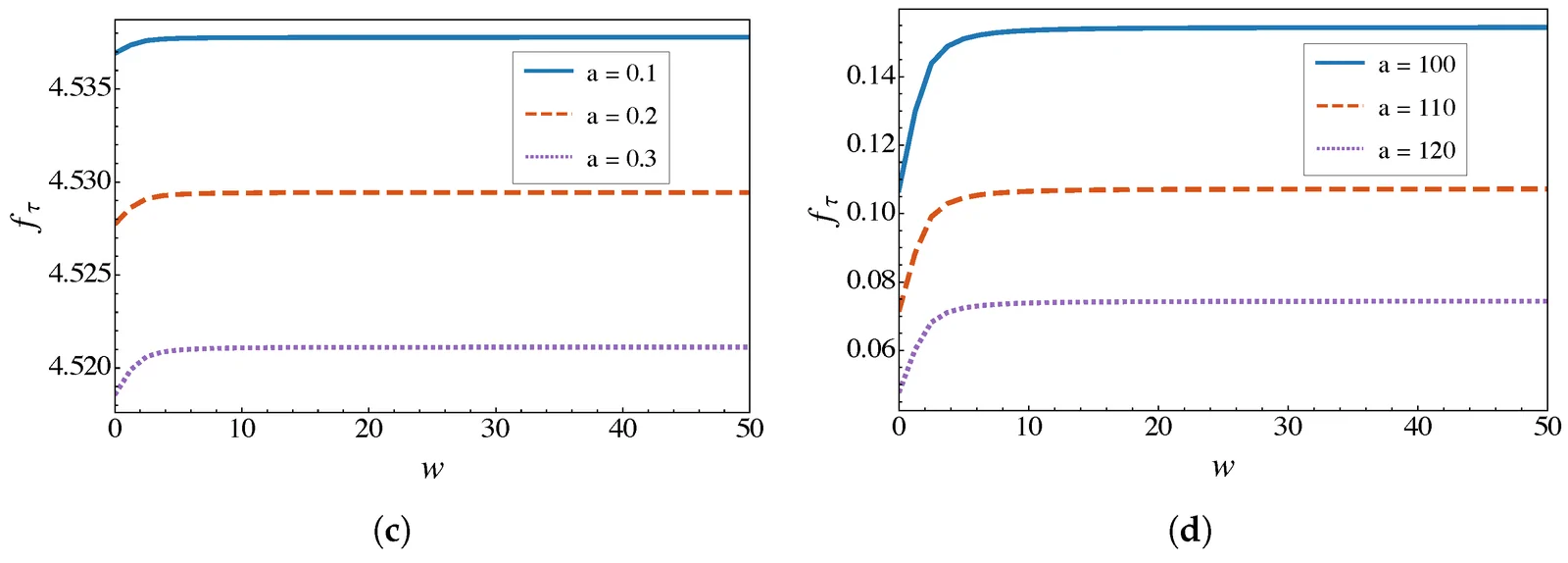
Dependence of $f_{\tau }$ on the four-velocity component *w* under different four-accelerations *a* and charging times τ for a single-qubit probe characterized by Ω=5, θ=π/3, ϕ=π, and λ=0.1. (**a**,**b**) $f_{\tau }$ versus *w* for small τ=0.001 with (**a**) small *a* and (**b**) increasing *a*. (**c**,**d**) $f_{\tau }$ versus *w* for τ=20 with (**c**) small *a* and (**d**) increasing *a*.

Figure 4 shows the precision of estimating the charging time τ in the quantum battery as a function of the transverse four-velocity *w* for different four-accelerations *a* and charging times.

For short charging times (Figure 4a,b), at lower values of *a*, the QFI decreases as *w* increases. As *a* is raised, the dependence changes and $f_{\tau }$ instead increases with *w*. Increasing the four-acceleration can therefore reverse the sign of the dependence of the charging-time QFI on the transverse four-velocity. For longer charging times (Figure 4c,d), the dependence on *w* is monotonic: $f_{\tau }$ increases with *w*.

In every case, $f_{\tau }$ approaches a constant value for large *w*. The value of this plateau and the range of *w* over which it is reached are not the same for all situations: both depend on the four-acceleration and on the charging time. What is common is only that the growth or decay of $f_{\tau }$ with *w* does not continue without bound: at sufficiently large *w*, any further increase of the transverse four-velocity changes $f_{\tau }$ only marginally.

Transverse motion has no fixed detrimental or beneficial effect on charging-time estimation: its effect depends on the acceleration and on the duration of the charging protocol. The metrological role of transverse motion is thus parameter dependent. This implies that the four-acceleration and the interaction time are not only resources for enhancing precision directly but also tools for selecting whether transverse motion acts in favor of or against the estimation of τ.

### 4.2. Two-Qubit Probe Estimation with an Isolated Ancilla

The density matrix of the two-qubit probe in Equation (37) exhibits a general non-X structure with off-diagonal elements that do not conform to the block-diagonal form commonly assumed in the literature. Consequently, closed-form analytic expressions for the quantum Fisher information are not readily available. Instead, we compute the QFI for each parameter separately using the diagonal elements of the quantum Fisher information matrix given in Equation (6). Specifically, $f_{\phi }=H_{11}$ and $f_{\tau }=H_{22}$ (with $\lambda_{1}=\phi$, $\lambda_{2}=\tau$). We emphasize that this does not constitute a simultaneous two-parameter estimation; the off-diagonal term $H_{12}$ is not used, and the individual QFIs are evaluated independently under the assumption that only the parameter of interest is unknown. The numerical evaluation is performed using the vectorized QFIM of Equation (6).

#### 4.2.1. Initial-Phase Estimation

**Figure 5:**
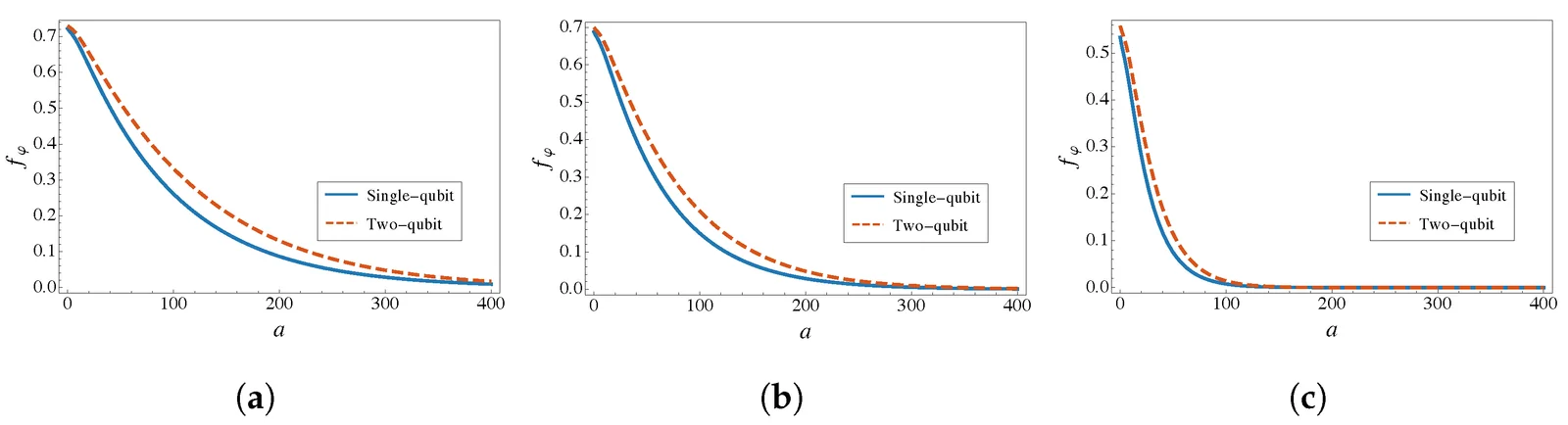
$f_{\phi }$ for phase estimation as a function of the four-acceleration *a* for a single-qubit probe and a two-qubit probe with an isolated ancilla in the ultra-relativistic regime with w=10, λ=0.1, Ω=5, θ=1.1, and ϕ=1 for charging times (**a**) τ=6, (**b**) τ=9, and (**c**) τ=25.

Figure 5 compares the phase QFI of the single-qubit probe with that of the two-qubit probe whose ancilla is isolated from the field for three charging times. At small and large values of the four-acceleration, the two quantities take comparable values, whereas at intermediate accelerations, the two-qubit QFI is larger. The advantage originates in the ancilla, which does not couple to the field and preserves part of the phase information that the interacting qubit loses through Unruh decoherence. At small *a*, this decoherence is too weak for the ancilla to affect the result, and at large *a*, it destroys the correlations that support the enhancement; the two-qubit QFI therefore exceeds the single-qubit QFI only over an intermediate range of accelerations.

For longer charging times, the interval of accelerations over which the two-qubit probe is advantageous shifts toward smaller values of *a*. Both ends of this interval move to weaker acceleration, since the accumulated decoherence grows with τ and the same loss of phase information is therefore reached earlier. Changing the transverse four-velocity does not modify this behavior.

**Figure 6:**
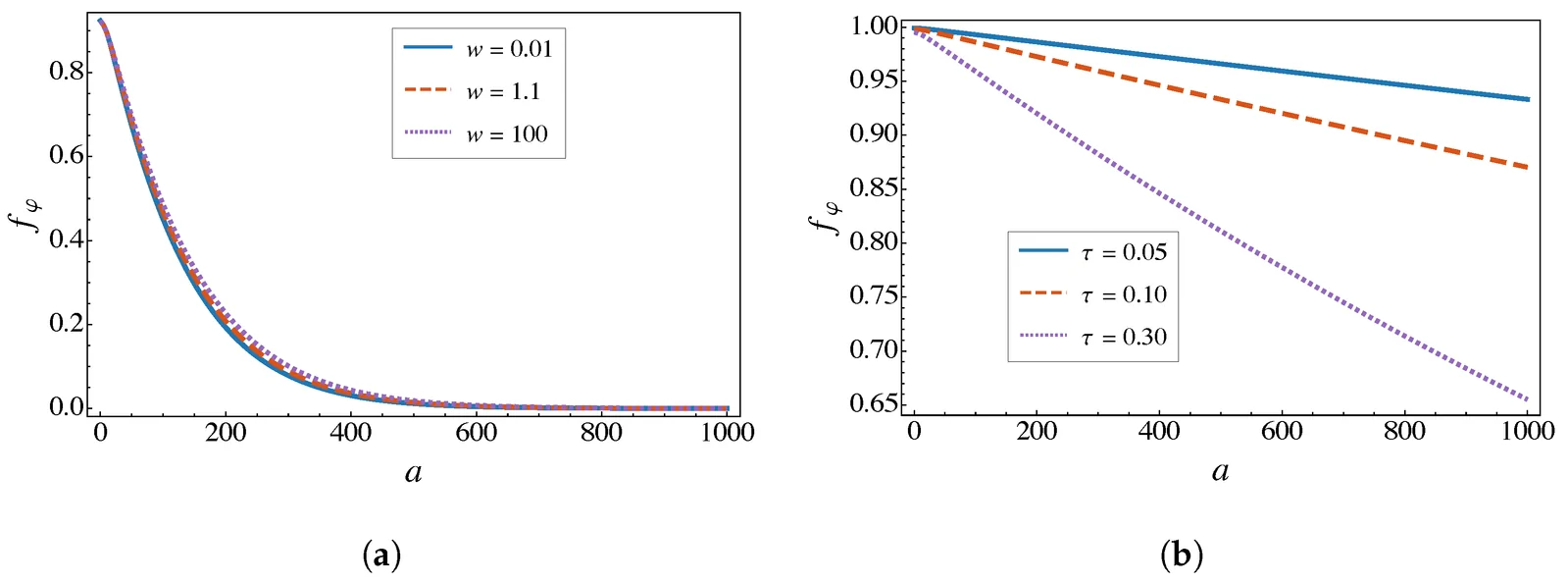
$f_{\phi }$ for phase estimation with the two-qubit probe. Common parameters for both panels are Ω=5 and λ=0.1. (**a**) $f_{\phi }$ versus four-acceleration *a* for different four-velocity components *w*, with τ=5, θ=π/2, and ϕ=2.3. (**b**) $f_{\phi }$ versus *a* for different charging times τ, with w=50, θ=π/2, and ϕ=1.

Figure 6 presents the phase QFI of the two-qubit probe. The dependence on the four-acceleration and on the transverse four-velocity follows the single-qubit case. Figure 6a shows that $f_{\phi }$ decreases with *a* for every transverse four-velocity *w*, and that the variation with *w* is weak. Figure 6b shows that the decrease of $f_{\phi }$ with *a* is steeper at longer charging times τ. This figure shows that the presence of the isolated ancilla does not modify the way in which $f_{\phi }$ depends on *a* and *w*. The two-qubit QFI differs in magnitude from the single-qubit result with the enhancement favoring the two-qubit probe at intermediate accelerations.

#### 4.2.2. Charging Time Estimation

**Figure 7:**
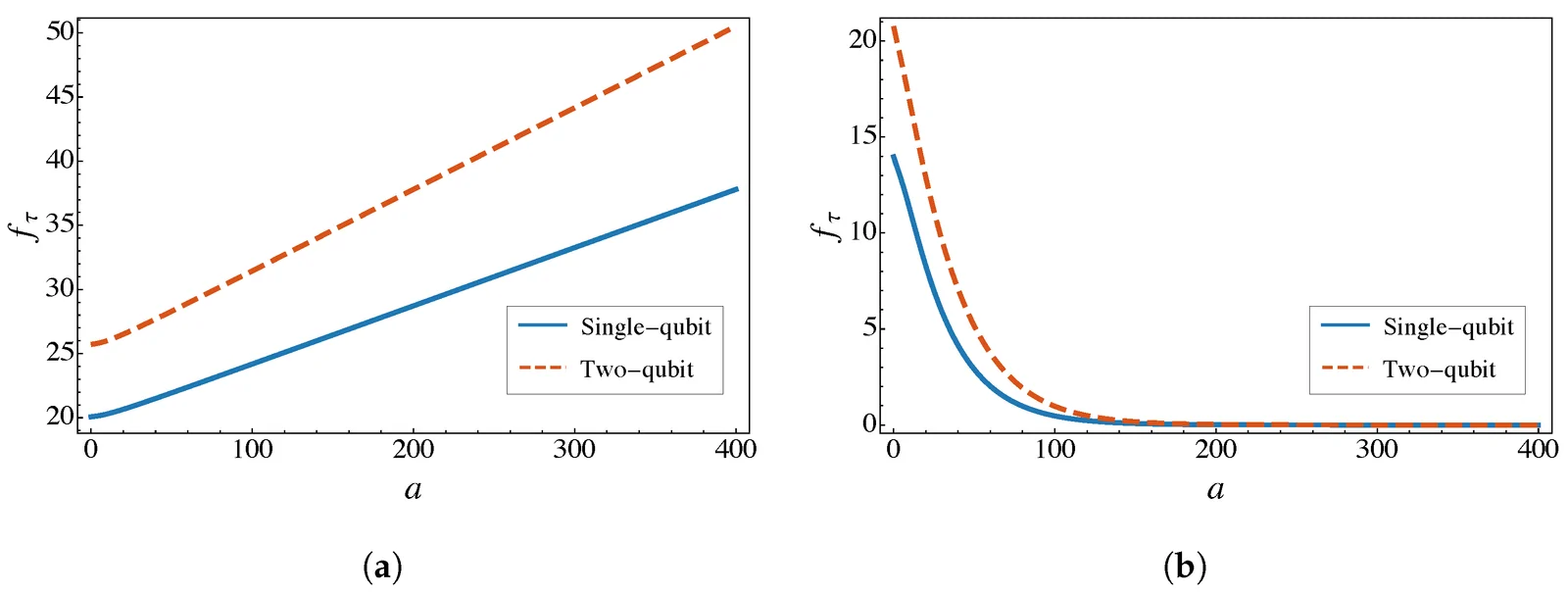
$f_{\tau }$ for a single-qubit probe (solid) and a two-qubit probe with an isolated ancilla (dashed) as functions of the four-acceleration *a*. Common parameters are w=10, Ω=5, θ=1.1, ϕ=1, and λ=0.1. (**a**) Short charging time, τ=0.01. (**b**) Long charging time, τ=20.

**Figure 8:**
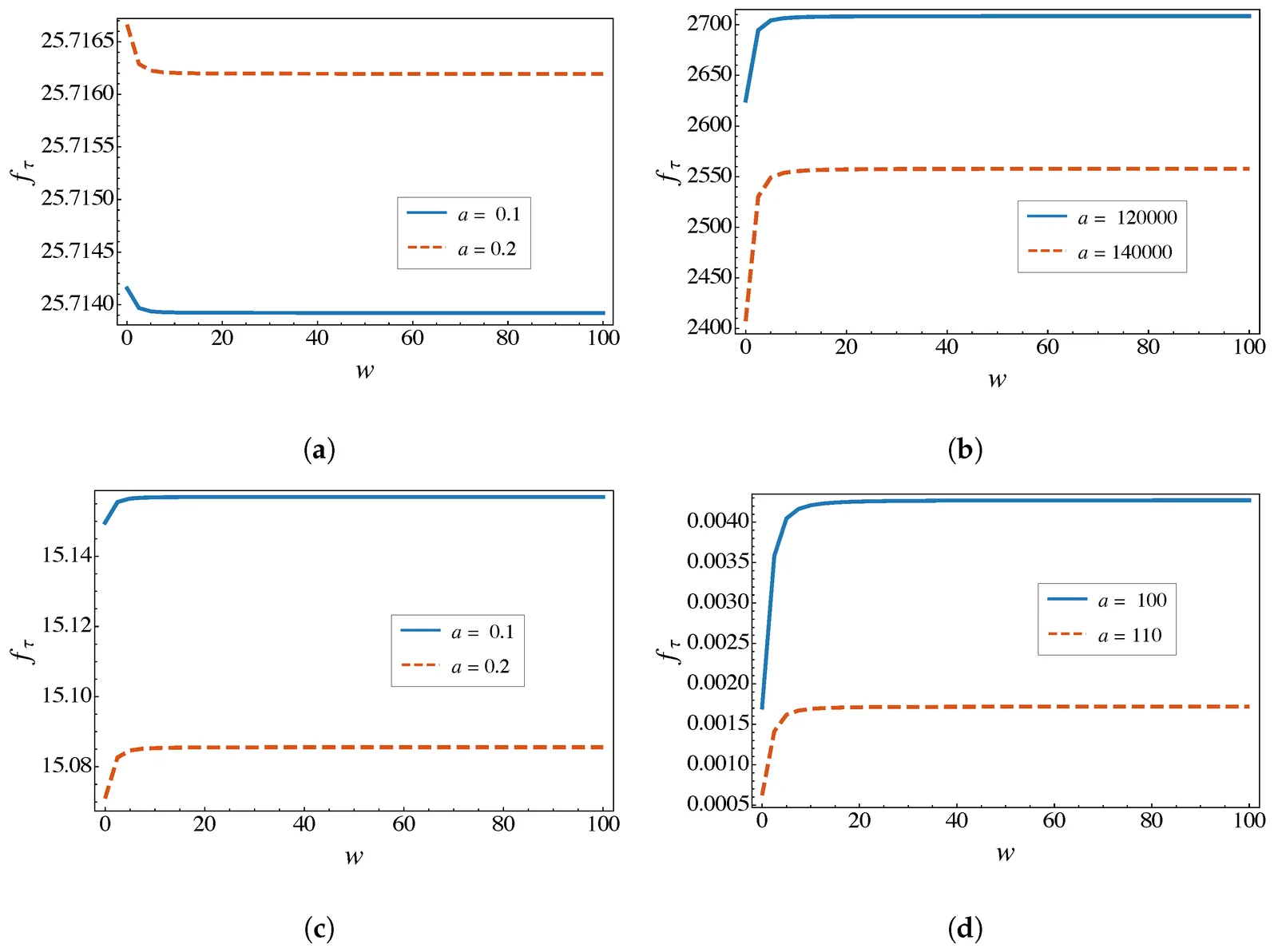
Dependence of $f_{\tau }$ on the four-velocity component *w* under different four-accelerations *a* and charging times τ for the two-qubit probe characterized by Ω=5, θ=1.1, ϕ=π, and λ=0.1. (**a**,**b**) $f_{\tau }$ versus *w* for small τ=0.01 with (**a**) small *a* and (**b**) increasing *a*. (**c**,**d**) $f_{\tau }$ versus *w* for τ=50 with (**c**) small *a* and (**d**) increasing *a*.

Figure 7 and Figure 8 report the charging-time QFI of the two-qubit probe. Figure 7 compares the single-qubit and two-qubit QFI directly, at a fixed transverse velocity, for a short and a long charging time. It shows that the presence of the isolated ancilla improves the precision with which the charging time can be estimated. The improvement is not the same at short and long τ: at long τ, it is concentrated at the lower accelerations, while at short τ, it persists over a wider interval. In both panels, the qualitative dependence on *a* is the same as that of the single-qubit probe: $f_{\tau }$ increases with *a* at short τ and decreases at long τ.

Figure 8 shows the corresponding dependence on the transverse four-velocity at a fixed charging time within each row. At short τ, the direction of the dependence is set by *a*: for small accelerations, $f_{\tau }$ decreases with *w*, whereas for larger accelerations, it increases. At long τ, $f_{\tau }$ increases with *w* for the accelerations shown. For large values of *w*, the QFI saturates. The qualitative behavior across the two figures matches that found for the single-qubit probe in Figure 3 and Figure 4.

The acceleration and the transverse velocity act on the battery alone. They change how the field appears to the detector and therefore how the duration of the drive is imprinted on the battery state. The ancilla is outside this dynamics: the field does not touch it, the drive does not act on it, and the trajectory does not affect it. Attaching it therefore does not alter how motion affects the estimation precision. What the ancilla changes is the initial condition on which the evolution acts. It begins entangled with the battery, so the same evolution acts on a correlated two-qubit state instead of a product state. This changes the amount of information that survives the drive but not the way the survival depends on the kinematics of the probe. The dependence of $f_{\tau }$ on acceleration and transverse velocity is a feature of the battery-field coupling, and this coupling is the same whether the ancilla is present or not.

The ancilla does not couple to the field, but it is not disconnected from the battery. Because the two qubits are initially entangled, the joint state remains correlated throughout the drive, so the degradation of the battery’s coherence under the field is reflected in the joint state even after the reduced state of the battery has become nearly stationary. A measurement on both qubits can therefore retrieve part of the information that the drive has imprinted on the battery and that the reduced state no longer carries. This is the origin of the two-qubit advantage over the single-qubit probe seen in Figure 7.

## 5. Quantum Correlations and Metrological Sensitivity

### 5.1. Entanglement Role

**Figure 9:**
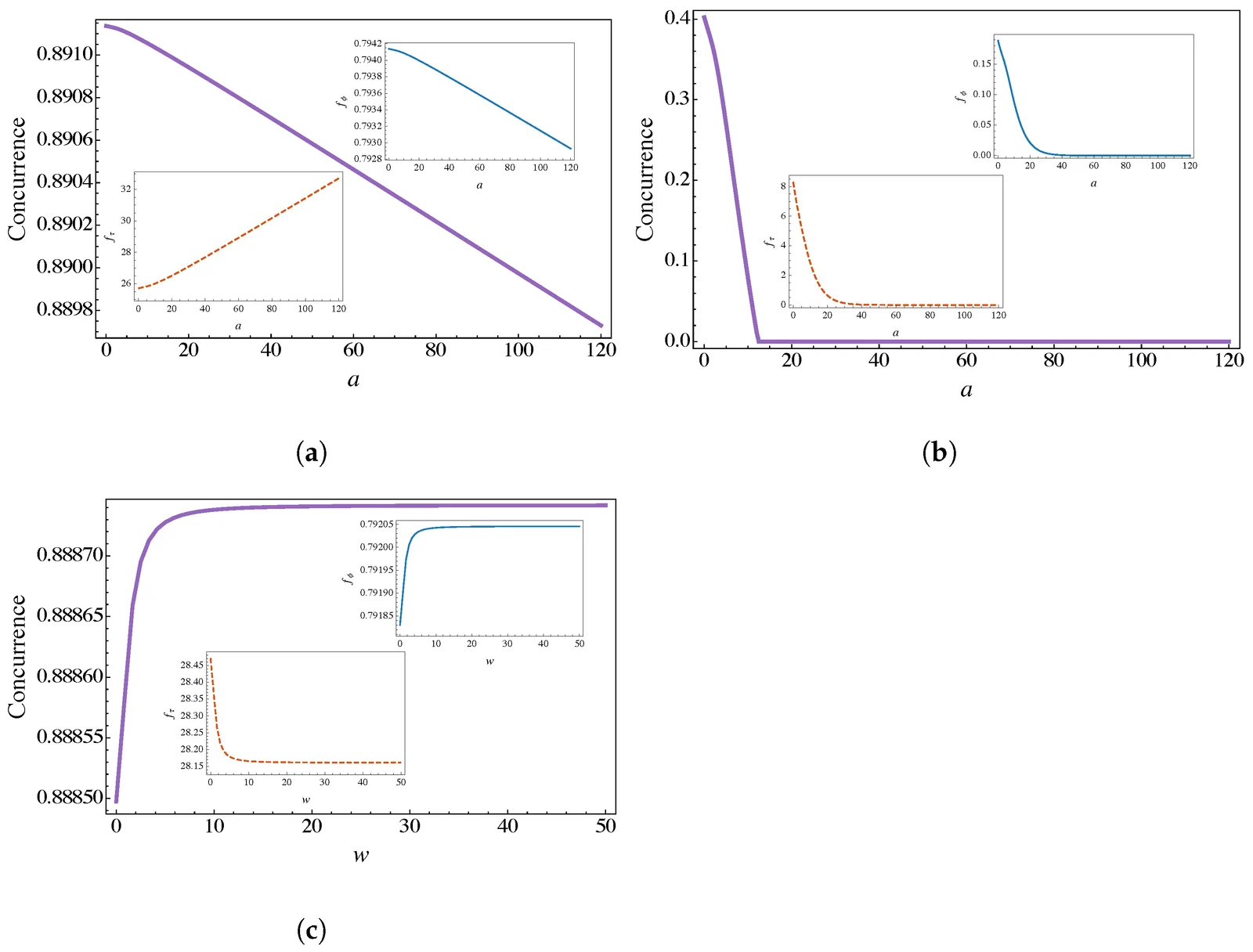
Comparison between the phase QFI $f_{\phi }$, the charging-time QFI $f_{\tau }$, and the concurrence of the two-qubit state. In each panel, the concurrence is the main curve and the two QFIs are shown in the insets on their own vertical scales. Common parameters are Ω=5, λ=0.1, θ=1.1, and ϕ=0. (**a**) Versus the four-acceleration *a* at small charging time τ=0.01 and w=50. (**b**) Versus *a* at large charging time τ=100 and w=50. (**c**) Versus the transverse four-velocity *w* at small charging time τ=0.02 and large four-acceleration a=100.

We first look at the role played by entanglement in the metrological performance of the two-qubit probe. Figure 9 compares the phase QFI, the charging-time QFI, and the concurrence of the two-qubit state. Panels (a) and (b) scan the four-acceleration at a short and a long charging time, and panel (c) scans the transverse four-velocity at a large acceleration.

The comparison shows that the qualitative behavior of the phase QFI closely follows that of the concurrence, whereas the charging-time QFI is not fully consistent with concurrence with deviations visible in panels (a) and (c).

A distinct feature appears when *a* is the scan variable. For the longer charging times considered, the numerical computations show that the concurrence reaches zero at a critical acceleration $a_{c}$, beyond which the two-qubit state is separable. Both QFIs remain nonzero beyond $a_{c}$ and decrease as *a* is increased further, approaching zero only gradually.

The survival of the QFIs beyond $a_{c}$ has a clear physical origin. The disappearance of concurrence marks the loss of entanglement between the two qubits, but it does not eliminate the dependence of the evolved probe state on the initial phase ϕ or on the charging duration τ. Even after entanglement has vanished, the separable probe can still carry finite metrological sensitivity. As *a* increases further, acceleration-induced dissipation progressively weakens this parameter encoding, and the QFIs decrease smoothly toward zero rather than dropping to zero at the entanglement-death point.

**Figure 10:**
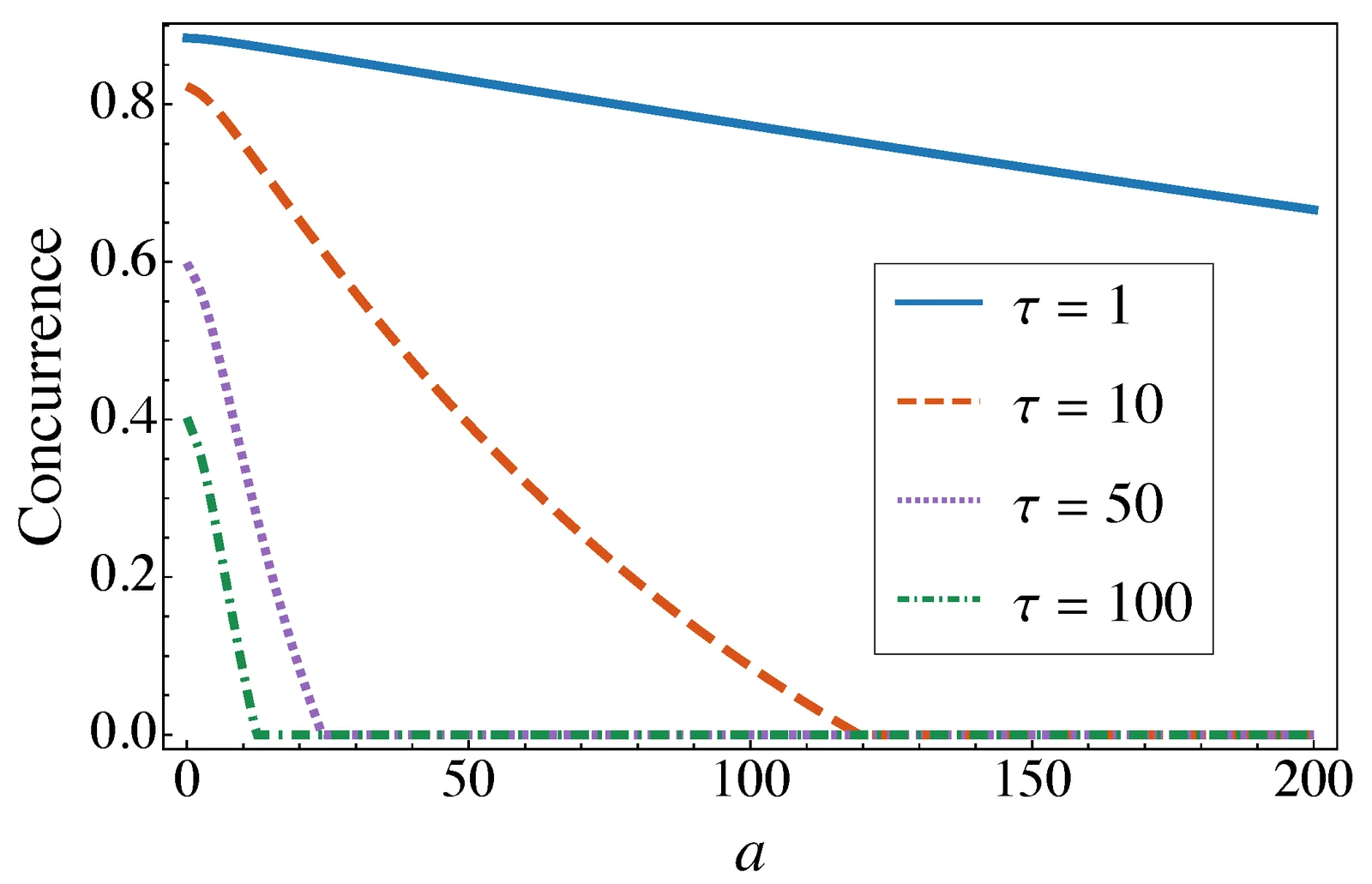
Concurrence of the two-qubit state as a function of the four-acceleration *a* for different charging times τ. The two-qubit state is prepared in the entangled state of Equation (34), only the battery qubit interacts with the field, and the ancilla remains isolated. Common parameters are Ω=5, λ=0.1, θ=1.1, ϕ=0, and w=10. The curves correspond to τ=1, 10, 50, and 100. The concurrence vanishes at a critical acceleration $a_{c}$ whose position depends on τ.

To see how this transition depends on the duration of the drive, Figure 10 shows that the critical acceleration at which the concurrence vanishes is not fixed. For the parameters shown, it decreases as the charging time is increased: a longer τ brings the two-qubit state to separability at a smaller acceleration.

Entanglement is progressively degraded by the field-induced open-system dynamics during the charging interval. Increasing τ gives this evolution more time to act, while changing *a* modifies the dissipative dynamics through the acceleration-dependent response coefficients. The combination produces, for the parameters considered here, the observed shift of the critical acceleration toward smaller values.

This has a direct consequence for the operating region of the two-qubit probe. At a fixed acceleration, reducing the charging time can preserve entanglement to larger accelerations, whereas longer charging times lead to an earlier loss of entanglement for the parameters shown. The shift of the critical acceleration with τ identifies a boundary in the *a*–τ plane that separates the region in which the state remains entangled from the region in which it has become separable.

### 5.2. Quantum Discord Role in Protecting Parameter Estimation

Having found the boundary beyond which entanglement is lost, we now ask whether nonclassical correlations of a different kind survive in the separable region and whether they accompany the residual metrological sensitivity of the probe.

**Figure 11:**
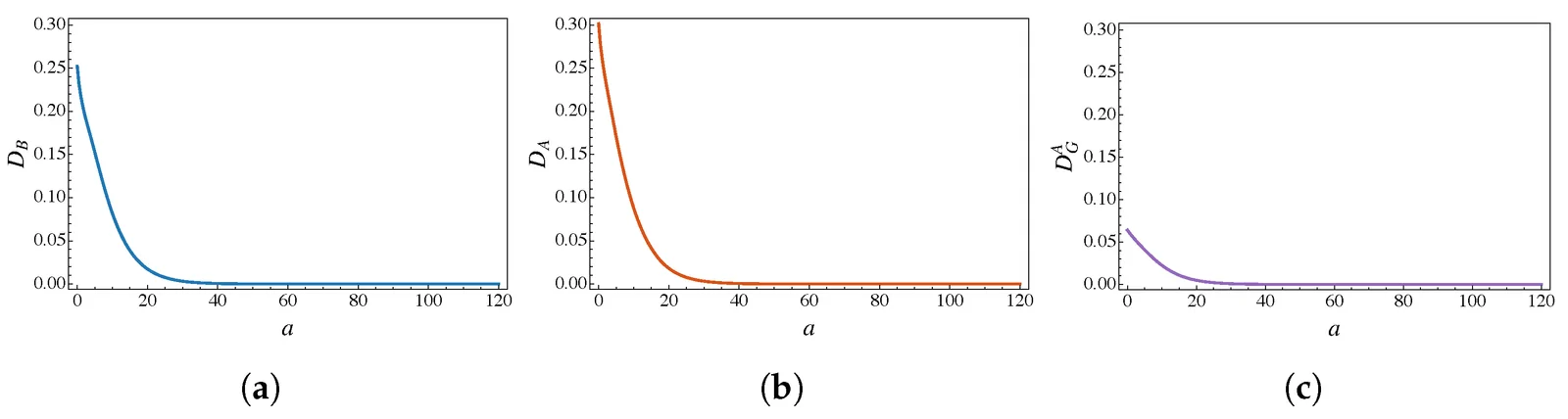
Quantum correlation measures of the two-qubit probe as functions of the four-acceleration *a*. (**a**) Projective-measurement entropic discord $D_{B}$ with the measurement on the isolated ancilla *B*. (**b**) Projective-measurement entropic discord $D_{A}$ with the measurement on the field-coupled qubit *A*. (**c**) *A*-side geometric discord $D_{G}^{A}$ of Equation (19). Common parameters are Ω=5, λ=0.1, θ=1.1, ϕ=0, w=50, and τ=100. All three measures decrease over the same interval of *a*. The two entropic discords differ slightly, reflecting the A↔B asymmetry of $\rho^{\left(2q\right)}\left(\tau \right)$. The three quantities are distinct functionals of the same state and have different numerical scales; they should not be identified numerically.

Figure 11 shows that the entropic discords $D_{A}$, $D_{B}$ and the geometric discord $D_{G}^{A}$ decrease over the same interval of *a* with similar qualitative behavior, which serves as a consistency check across the three measures. In the two-qubit probe, only one qubit interacts with the field. The Minkowski vacuum provides a trajectory-dependent open-system environment whose response depends on both the four-acceleration *a* and the transverse four-velocity *w*, while the ancilla remains dynamically isolated from the field. This one-sided coupling makes the joint state asymmetric under exchange of the two qubits, which is why the two entropic discords differ slightly from one another.

**Figure 12:**
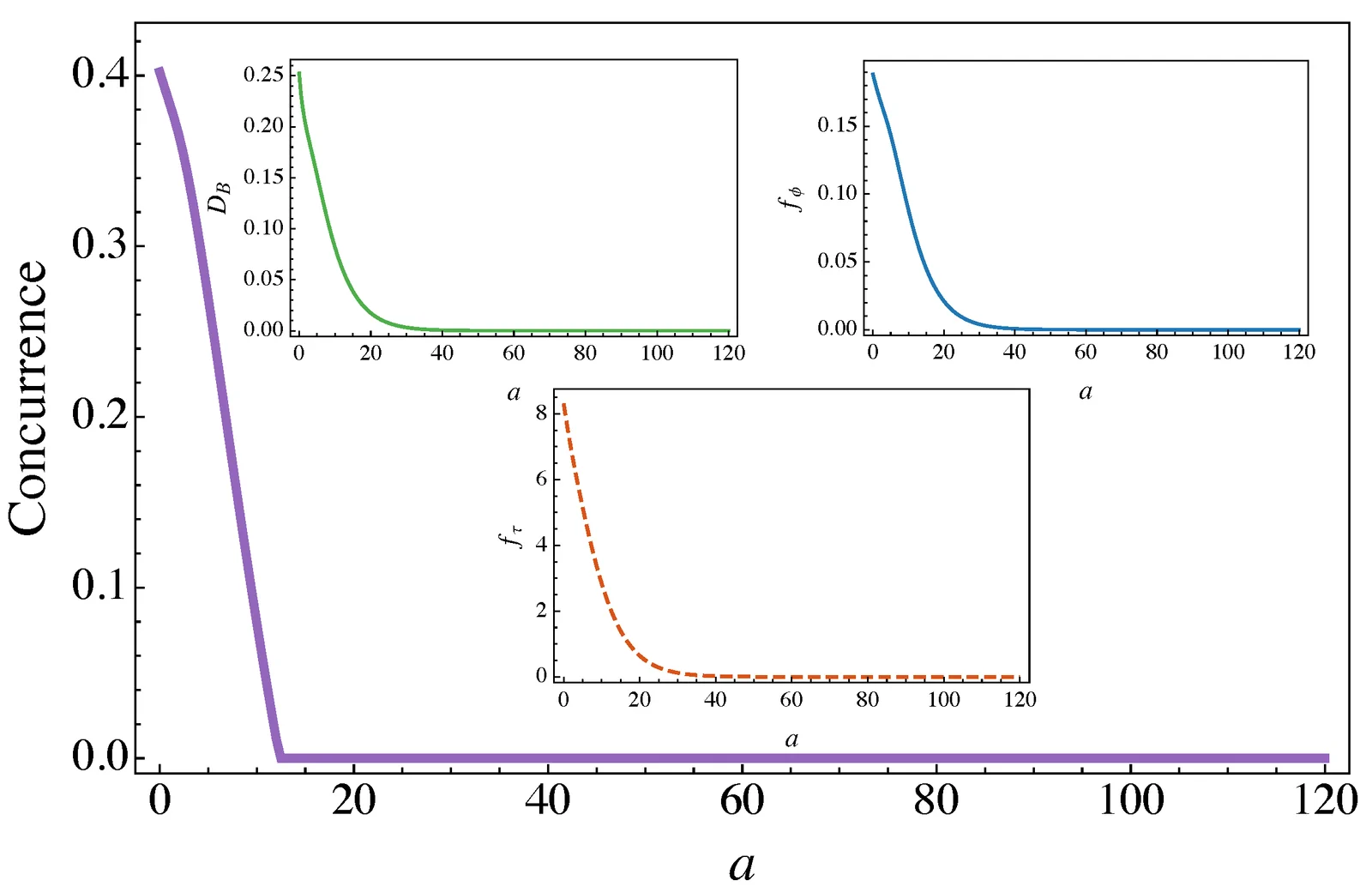
Concurrence, discord, and quantum Fisher information of the two-qubit probe as functions of the four-acceleration *a*. Main curve: Wootters concurrence, vanishing at the critical acceleration $a_{c}$, marking the transition to a separable state. Top-left inset: projective-measurement entropic discord $D_{B}$, which remains nonzero over the resolved acceleration range shown. Top-right inset: quantum Fisher information for the initial phase. Bottom-right inset: quantum Fisher information for the charging time. Both QFIs remain finite beyond $a_{c}$ and decrease smoothly as *a* grows, following the same qualitative behavior as the discord. Parameters as in Figure 11.

Figure 12 compares the three correlation measures with the concurrence and with the quantum Fisher informations (QFIs). At a critical acceleration, the concurrence vanishes and the state becomes separable, yet the discord stays nonzero, since it detects nonclassical correlations that a separable state can still carry. The two QFIs remain finite beyond this threshold and decrease together with the discord over the acceleration range shown, tending toward zero as *a* is increased. The figures therefore show a co-variation between the residual discord and the residual metrological sensitivity of the probe past the separability point. This behavior is consistent with earlier studies showing that discord-type correlations or coherence can persist beyond entanglement loss and retain metrological relevance [58,60,68,69]. Direct evidence for residual quantum correlations in accelerating Unruh–DeWitt detectors has also been reported [70]. Whether this co-variation reflects a general feature of relativistic probes or is specific to the trajectory and initial state considered here is left for future investigation.

## 6. Conclusions

We have studied quantum parameter estimation for a relativistically moving Unruh–DeWitt probe whose reduced state is not restricted to the X-state form used in earlier analytical work. Two parameters were considered: the initial phase encoded in the probe before the drive and the charging time of the drive itself. The single-qubit case was compared with a two-qubit case in which an isolated ancilla is entangled with the field-coupled qubit at the start of the evolution.

For a single qubit, the two parameters respond to acceleration in different ways. The phase QFI decreases as the acceleration grows, and the decrease is larger at longer drive durations. The charging-time QFI instead increases with acceleration at short drive durations, and this increase is gradually replaced by a decrease as the drive duration becomes longer.

For the two-qubit probe, the isolated ancilla leads to a higher phase QFI than the single-qubit probe at intermediate accelerations. The advantage comes from the ancilla, which preserves part of the phase information that the interacting qubit loses through Unruh decoherence. As the drive duration increases, the interval of accelerations over which the two-qubit probe is advantageous shifts toward smaller accelerations. The transverse velocity of the probe has little effect on this behavior. For charging-time estimation, the ancilla also improves the precision; the improvement is concentrated at lower accelerations for long drive durations, and it persists over a wider interval at short drive durations. The dependence of the charging-time QFI on acceleration and transverse velocity follows the same behavior as in the single-qubit case.

The behavior of the two parameters is not uniform with respect to the concurrence of the two-qubit state. The phase QFI decreases in a manner similar to the concurrence, whereas the charging-time QFI does not follow the same pattern at short drive durations. This difference indicates that conclusions about quantum resources drawn from entanglement measures do not extend uniformly across metrological parameters.

The resemblance between the phase QFI and the concurrence is qualitative and does not extend to their vanishing points. The concurrence reaches zero at a critical acceleration, beyond which the two-qubit state is separable. Both QFIs remain nonzero beyond this point, and the discord of the reduced state also remains nonzero. We found that in our model, the two-qubit QFIs decrease together with the discord. These results point to quantum correlations beyond entanglement as a resource for relativistic parameter estimation in the separable regime.

The present results can be compared with the literature on X-state relativistic quantum systems. Accelerated X-state probes have displayed parameter-dependent Fisher-information behavior, including intervals of constant Fisher information [57]. More recently, phase-encoded X states have been shown to retain finite phase quantum Fisher information after entanglement sudden death [60]. The present analysis considers instead a finite-time driven Unruh–DeWitt dynamics in which one qubit interacts with the scalar field while an ancillary qubit remains isolated, yielding a general non-X reduced state. Accordingly, the persistence of metrological sensitivity after entanglement loss is not limited to X-state systems but also occurs in the more general non-X case.

Estimating the charging time through the quantum Fisher information can be related to quantum speed limits [71], which set fundamental bounds on how rapidly a quantum state may evolve under a given dynamics. Entanglement has been shown to raise this bound above the limit attainable by separable states [72]. The charging-time QFI quantifies how precisely the drive duration can be inferred from the final probe state, while a quantum speed limit constrains the minimal time required for that evolution. In the relativistic model considered here, examining the interplay between these two quantities constitutes a pertinent extension, particularly in view of recent advances in relativistic quantum information [73,74]. This also connects to the broader set of open questions. The role of the initial-state parameters, the effect of non-Markovian corrections at stronger coupling, the extension to three-dimensional trajectories, and the analysis of joint estimation of both parameters together are natural next steps [75,76]. The framework introduced here can be adapted to other trajectories and detector configurations.

## Data Availability

The original contributions presented in this study are included in the article. Further inquiries can be directed to the corresponding authors.

## Abbreviations

The following abbreviations are used in this manuscript:

QFI	quantum Fisher information
QFIM	quantum Fisher information matrix
